# Gene expression analysis reveals a 5-gene signature for progression-free survival in prostate cancer

**DOI:** 10.3389/fonc.2022.914078

**Published:** 2022-08-12

**Authors:** Zhuofan Mou, Jack Spencer, Bridget Knight, Joseph John, Paul McCullagh, John S. McGrath, Lorna W. Harries

**Affiliations:** ^1^ Institute of Biomedical and Clinical Sciences, University of Exeter Medical School, Devon, United Kingdom; ^2^ Translational Research Exchange at Exeter, Living Systems Institute, University of Exeter, Exeter, United Kingdom; ^3^ National Institute for Health and Care Research (NIHR) Exeter Clinical Research Facility, Royal Devon and Exeter National Health Service (NHS) Foundation Trust, Royal Devon and Exeter Hospital, Exeter, United Kingdom; ^4^ Exeter Surgical Health Services Research Unit, Royal Devon and Exeter National Health Service (NHS) Foundation Trust, Exeter, United Kingdom; ^5^ Department of Pathology, Royal Devon and Exeter National Health Service (NHS) Foundation Trust, Exeter, United Kingdom

**Keywords:** gene signature, prostate cancer, transcriptomics, prognosis, prediction model

## Abstract

Prostate cancer (PCa) is the second most common male cancer worldwide, but effective biomarkers for the presence or progression risk of disease are currently elusive. In a series of nine matched histologically confirmed PCa and benign samples, we carried out an integrated transcriptome-wide gene expression analysis, including differential gene expression analysis and weighted gene co-expression network analysis (WGCNA), which identified a set of potential gene markers highly associated with tumour status (malignant *vs*. benign). We then used these genes to establish a minimal progression-free survival (PFS)-associated gene signature (GS) (*PCBP1*, *PABPN1*, *PTPRF*, *DANCR*, and *MYC*) using least absolute shrinkage and selection operator (LASSO) and stepwise multivariate Cox regression analyses from The Cancer Genome Atlas prostate adenocarcinoma (TCGA-PRAD) dataset. Our signature was able to predict PFS over 1, 3, and 5 years in TCGA-PRAD dataset, with area under the curve (AUC) of 0.64–0.78, and our signature remained as a prognostic factor independent of age, Gleason score, and pathological T and N stages. A nomogram combining the signature and Gleason score demonstrated improved predictive capability for PFS (AUC: 0.71–0.85) and was superior to the Cambridge Prognostic Group (CPG) model alone and some conventionally used clinicopathological factors in predicting PFS. In conclusion, we have identified and validated a novel five-gene signature and established a nomogram that effectively predicted PFS in patients with PCa. Findings may improve current prognosis tools for PFS and contribute to clinical decision-making in PCa treatment.

## 1 Introduction

Prostate cancer (PCa) is the second most common malignancy in men worldwide, with an estimated 1.41 million new cases and over 375,000 deaths in 2020 (1). Predicting the risk of treatment failure is important because this can inform whether to use neoadjuvant or adjuvant therapies (2) and how closely patients should be followed up. Risk of treatment failure has conventionally been calculated using clinical (T-stage), biochemical [prostate-specific antigen (PSA)], and histological (Gleason score) parameters (3), and the prognostic power of these clinicopathological features is often limited, such as in patients with ambiguous clinical diagnoses. Improvements on these models could be achieved by identifying novel biomarkers, and the use of reliable biomarkers might assist in the determination of the prognosis and, therefore, the success of the treatment (4).

Previous studies have used differential gene expression to differentiate aggressive and indolent PCa subtypes using transcriptome-wide technologies (5, 6). There is sometimes little overlap between the gene sets identified, and comparison between benign and malignant tissues deriving from different subjects can introduce biological noise. To date, reliable signatures of aggressive PCa are scarce (7), and there is a clear need for more precise molecular diagnostic and prognostic markers.

In order to improve the accuracy of the prognosis in PCa, a variety of survival-related molecular biomarkers have been identified and validated to predict disease progression. For example, three eRNA-driven genes were identified to predict disease-free survival (DFS) (8), a three-gene based signature was developed for predicting both overall survival (OS) and DFS (9), and a six-gene signature associated with castration-resistant PCa was established to predict recurrence-free survival (RFS) (10). Previous studies have identified a few progression-related biomarkers, such as a 49-gene signature specific for predicting metastatic-lethal (ML) progression (11), a two-gene signature for predicting circulating tumour cell (CTCs) levels (12), and various biomarkers built from a binary classifier that can predict different pairs of PCa progression stages/substages (13). However, all of these progression-associated biomarkers and many others (14) were selected and modelled based on two- or multi-level classifiers, which do not take progression-free survival (PFS) time and survival status data (i.e., progressed or not progressed) into account, and therefore are unable to predict PFS.

In this study, we aimed to produce transcriptome-wide expression profiles from a series of nine PCa and matched benign samples from the same individuals to determine differentially expressed genes (DEGs) and co-expression modules. The most representative and highly correlated DEGs were integrated with the hub genes identified through weighted gene co-expression network analysis (WGCNA) and protein–protein interaction (PPI) network analysis to be considered as our candidate genes. We then used the candidate genes in conjunction with an external dataset [The Cancer Genome Atlas prostate adenocarcinoma (TCGA-PRAD)] to produce a minimal 5-gene expression signature (*PCBP1*, *PABPN1*, *PTPRF*, *DANCR*, and *MYC*) using the least absolute shrinkage and selection operator (LASSO) and Cox regression methods to predict PFS. Kaplan–Meier (KM) method and log-rank test were used to test the association between each signature gene and PFS of PCa. The gene signature was then combined with PCa-associated clinical parameter(s) to define a nomogram for PFS. Finally, we compared the clinical utility of our gene signature and nomogram in combination with and against established prediction tools such as the Cambridge Prognostic Group (CPG) model and other commonly used clinical features in PCa for the prediction of PFS. Overall, we found that both our gene signature and nomogram were capable of predicting PFS of patients with PCa, and thus, may have potential clinical utility as prognostic tools in the future.

## 2 Materials and methods

### 2.1 Patient samples

Benign and matched PCa tissue was obtained using radical prostatectomy specimens from nine patients. Histologically confirmed areas of PCa and benign regions within the same prostate sample were excised and stored in RNAlater (Thermo Fisher, Waltham, MA, USA) prior to RNA extraction. The samples were obtained with written consent and ethical approval through the Exeter NIHR Clinical Research Facility tissue bank (Ref: STB20). Patient characteristics are given in **Table 1**.

**Table 1:** Patient characteristics of the paired Clariom D sample set.

Patient number	Age	Gleason score	TNM score	Smoking status	Family history
007 RP	66	4+3+5	pT3aN1	No	No
008 RP	67	4+3	pT2cN0	No	No
009 RP	65	4+3+5	pT3aN0	Yes	Father with breast cancer
010 RP	53	3+4	pT3aNX	No	No
012 RP	64	4+5+3	pT3aN0	Former smoker (17 years ago)	No
013 RP	59	3+4	pT2NX	No	No
014 RP	67	4+5	pT3bN0	Yes	No
015 RP	70	4+3+5	pT2cN0	No	Father with prostate cancer
017 RP	67	3+4	pT2cN0	Former smoker (6 weeks ago)	Strong family history of bowel cancer

TNM: T, tumour size; N, lymph node status; M, metastasis.

### 2.2 RNA extraction

Tissue samples preserved in RNAlater were thawed and minced using a scalpel. RNA was then extracted using the Qiagen blood mini kit (Hilden, Germany), adapted by the use of a Qiashredder column (Hilden, Germany) on the Qiacube platform (Hilden, Germany). RNA concentration and integrity were assessed using the Agilent Bioanalyser 2100 platform (Agilent, Santa Clara, CA, USA).

### 2.3 Transcriptomic profiling and array data preprocessing

Gene expression was measured on the Clariom D Pico GeneChip Whole Transcriptome (WT) platform (Thermo Fisher, Waltham, MA, USA). RNA integrity was assessed as part of the library preparation (UK Bioinformatics, King’s College, London, UK). Data underwent quality control for probeset mean for hybridisation intensity, probeset residual mean that compares probeset signal to residual signal, poly-A-positive spike in controls as control genes and positive vs. negative area under the curve.

To preprocess the raw Clariom D dataset, the R package *oligo* (15) was used along with the Robust Multichip Average (RMA) algorithm (16), which summed up 138,745 captured transcripts. Transcripts were aligned to build 37 (GRCh37/hg19) of the human transcriptome using the BioConductor Platform Design (pd) package *pd.clariom.d.human*. Annotation was performed using the R package *affycoretools* (17), giving a total of 86,161 annotated transcripts (~62%). The dataset was then filtered to remove transcripts with mean intensities less than the threshold in less than 50% of the samples. Unannotated and uninformative probes (i.e., probes without a gene name or identifier; probes mapped to multiple genes, pseudogenes, and those predicted by the 'AceView' database) were also filtered out. Multiple probes for the same gene were then collapsed by taking the probe with the highest average across all samples. We obtained a final set of 22,165 genes.

### 2.4 Identification of differentially expressed genes

DEGs between matched benign and malignant prostate tissue samples were identified using the R package *Limma* (18) to fit a linear model and an empirical Bayes moderated t-test applied for each gene. A Benjamini–Hochberg (BH) multiple hypothesis testing correction was applied to adjust for false discovery rate (FDR). DEGs were defined based on raw p-value<0.001, adjusted p-value<0.1, and |log2 fold change (FC)| >0.585. Non-parametric multivariate BIO-ENV analysis was then applied using PRIMER-Є software (version 6.1.18) (19). We measured (dis)similarity for the genes and used Spearman’s rank correlations between subset matrices and between-sample matrix (both in Euclidean distance) to identify the most representative gene subsets capturing the total DEGs for benign *vs*. malignant tumour comparison. Subsets with p-value <0.05 were considered significant.

### 2.5 Weighted gene co-expression network analysis

WGCNA was conducted on the 22,165 genes using the R package *WGCNA* (20, 21). The minimum soft-thresholding power satisfying the scale-free fit index of 0.80 was selected. The module detection was constructed with a dynamic merging branch cut of 0.25 in a signed network design. Topological overlap matrix (TOM) dissimilarity plots were used to visualise the gene network structure. Each module was summarised by an eigengene (ME, the first principal component of all co-expressed genes in the module). To identify key module(s) of interest, Pearson correlation was applied between the MEs and tumour status (malignant or benign). Moreover, gene significance (GS; the Pearson correlation between genes and tumour status of a given module) and module significance (MS; the average of GS of all genes within a module) were used to verify the module–trait association(s).

### 2.6 Identification of module hub genes

Hub genes within a module were detected using two measures: 1) TOM-based intramodular connectivity and 2) based on module membership (MM; correlation between gene expression values and ME of a particular module). We first calculated the MM of each key module and then assessed MM *vs*. GS, MM *vs*. TOM-based intramodular connectivity, and GS *vs*. TOM-based intramodular connectivity to investigate the properties of the genes within each key module. The selection of module hub genes was based on the overlapped genes of the top 5% highest intramodular connectivity score of both TOM- and MM-based measures. Hub genes satisfied the thresholds of the absolute value of the MM >0.90 and GS >0.30. The Search Tool for Retrieval of Interacting Genes (STRING) was used to construct a PPI network prediction of module hub genes (22). Full STRING network with score confidence >0.4 and FDR<0.05 was applied on the hub gene set(s). Genes satisfying the thresholds were further analysed using Cystoscape (23). The Maximal Clique Centrality (MCC), an algorithm from the plugin CytoHubba (24), was used to rank gene connectivity in the respective PPI network. Lastly, the top 5 hub genes according to the MCC scores were selected from each module.

### 2.7 Pathway enrichment analysis

The R package *clusterProfiler* (25) was used to perform Gene Ontology (GO) and Kyoto Encyclopedia of Genes and Genomes (KEGG) pathway enrichment analyses on the hub genes within each key module associated with tumour status. GO enrichment analysis was performed primarily to identify biological process (BP), cellular component (CC), and molecular function (MF) associated with the identified gene sets, while KEGG analysis revealed their associations to biological pathways. We set FDR-adjusted p-value <0.05 as a cutoff criterion for significant enrichment.

### 2.8 Preprocessing of public sequencing data and validation of gene expression

For prognostic model development, raw RNA-seq counts and clinical, genetic, pathological, and radiological data from TCGA-PRAD were obtained using the R package *TCGAbiolinks* (26). Raw count data were preprocessed using the R package *edgeR* (27–29), and the corresponding progression-free interval (PFI) and status information of each sample were downloaded from the UCSC Xena PAN-Cancer database (https://xenabrowser.net; version: 2018-09-13). PFS was used as the primary clinical endpoint as the most reliable outcome for PCa (30) and was defined as the interval between the date of diagnosis and the date of the new event returned, including the progression of the cancer, local recurrence, distant metastases, or death from the cancer.

Differential expression of candidate genes was then validated using TCGA-PRAD dataset. For model construction and validation, we eliminated one metastatic tumour sample, all normal tissue samples, and three samples with follow-up less than 1 month, resulting in a total of 492 tumour cases. Moreover, the samples were randomly divided into a training set (n = 345; 70%) and a testing set (n = 147; 30%). The progression event and clinicopathological characteristics of the two internal sets are summarised in **Table 2**.

**Table 2:** Clinical characteristics of TCGA-PRAD sets of the prostate cancer tumour samples for progression-free survival (PFS) analysis.

Clinical feature	Training set	Validation set
	TCGA-PRAD (n = 345)	TCGA-PRAD (n = 147)
Age (years) (%)		
<60	141 (40.9)	59 (40.1)
≥60	204 (59.1)	88 (59.9)
Progressed events (%)	65 (18.8)	28 (19.0)
Pathological T stage (%)		
T2	121 (35.1)	66 (44.9)
T3–T4	220 (63.8)	79 (53.7)
Unknown	4 (1.2)	2 (1.4)
Pathological N stage (%)		
N0	237 (68.7)	105 (71.4)
N1	57 (16.5)	21 (14.3)
Unknown	51 (14.8)	21 (14.3)
Gleason score (%)		
≤7	200 (58.0)	90 (61.2)
>7	145 (42.0)	57 (38.8)

TCGA, The Cancer Genome Atlas; PRAD, prostate adenocarcinoma; Age, age at diagnosis; T stage, tumour stage; N stage, lymph node status (N0 = without lymph node metastasis; N1 = with lymph node metastasis); Unknown, missing data.

### 2.9 Prognostic model construction for progression-free survival of prostate cancer patients

LASSO regression analysis (31) was conducted using the R package *glmnet* (32) to narrow down the candidate genes in TCGA-PRAD training set. Subsequently, the multivariate Cox proportional hazards model was applied using the R package *survival* (33, 34) with a bidirectional stepwise variable selection procedure for the optimal gene combination that calculated the lowest Akaike information criteria (AIC) value. Finally, the prognostic risk model of each patient was defined as follows:


, $$survival\;risk\;score\;of\;each\;patient\;=\sum_{i=1}^{n}\left(coefficient_{i}\;\times \;gene\;expression_{i}\right)$$

where the **coefficient*_i_*** is the corresponding coefficient from the multivariate Cox regression of **gene*_i_*** and **gene expression *_i_*** is the expression value of **gene*_i_*.**

The predicted risk scores were classified into high and low risk using the function *surv_cutpoint* from the R package *survminer* (35) to find an optimal separation point. Finally, we assessed the significance of the difference between the two groups using KM and log-rank tests. Both training and testing sets were evaluated to determine the performance of the derived gene-based risk model. R package *rms* (36) was used to test the discrimination of our model using Harrell’s concordance index (C-index). Bias-corrected calibration analysis with 1,000 bootstrap sample permutations was used to check the consistency between predicted and observed probabilities for 1-, 3-, and 5-year PFS. Time-dependent receiver operating characteristic (ROC) analysis was conducted using the R package *survivalROC* (37) to assess the model accuracy according to the area under the ROC curve (AUC) for 1-, 3-, and 5-year PFS.

### 2.10 Independent prognostic ability of the signature and nomogram construction

Age, pathological stage T (tumour stage), N (lymph node status), and Gleason score are common clinicopathological factors in PCa and were accounted for assessing the prognostic power of our five-gene signature (**Table 2**). Univariate and multivariate Cox regression analyses were performed in the two TCGA-PRAD sets to assess the association between the input factors and PFS. Significant factors from the multivariate Cox analysis were selected to construct a nomogram for predicting 1-, 3-, and 5-year PFS. The calibration plot, ROC analysis, and C-index were used to evaluate the characteristics of the nomogram. Furthermore, we evaluated the clinical utility of our gene signature and nomogram through comparison with the well-established CPG model for predicting PCa PFS. The CPG model comprises the Gleason score, the PSA level, and the T stage. An algorithm was adopted to compute the CPG score according to Gnanapragasam et al. (38), but with pathological T stage rather than clinical T stage. We also evaluated the models against the Gleason score, pathological T stage, and serum PSA level (divided into three categories: <10 ng/ml, 10–20 ng/ml, and >20 ng/ml).

### 2.11 Statistical analysis

This study was conducted using the R software (version 4.0.5), PRIMER-Є software (version 6.1.18), and Cytoscape (version 3.8.2). For two-group differential analysis, the Student’s t-test was applied for the risk score-associated comparisons, and the Wilcoxon rank-sum test was applied for gene expression-associated comparisons. KM method and log-rank test were used to assess associations between variables and PFS. Statistical significance was set at p = 0.05 throughout this study, unless otherwise indicated.

## 3 Results

### 3.1 Differential gene expression in prostate cancer samples

**Figure 1:**
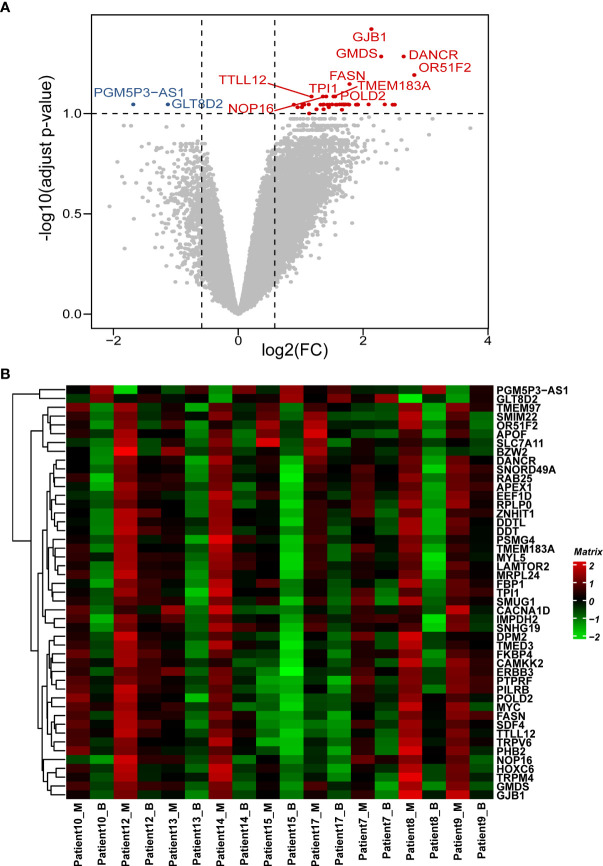
Patterns of differential expression in paired PCa samples. **(A)** Volcano plot of the DEGs between paired benign and malignant prostate samples. This graph is formed by plotting the log2(fold change) on the x-axis and calculating the -log10(adjusted p-value) along the y-axis. The red dots indicate genes that are upregulated (with labelled names), the blue dots indicate genes that are downregulated (with labelled names), and the black dots represent genes that are stable. The top 10 dysregulated genes were labelled. **(B)** Heatmap of the DEGs between the benign and malignant prostate samples. The genes are clustered vertically. Using the colour legend bar on the right-hand side, red represents high gene expression, green represents low gene expression, and black represents no gene expression. The expression values range from -2 to 2. DEG, differentially expressed gene. PCa, prostate cancer; M, malignant; B, benign.

In this study, 22,165 genes were expressed in human prostate samples after quality control (QC). We identified 47 DEGs under the cutoffs raw p-value <0.001, FDR-adjusted p-value <0.1, and |log2 FC| >0.585 (**Table S1** **;** **Figures 1A, B** **)**. The minimal gene set capturing the information in the complete DEG dataset was identified using BIO-ENV analysis. The top 5 subsets from the analysis are shown in **Table S2**, and the most significant gene set was defined by genes *PTPRF*, *MRPL24*, *DANCR*, *MYC*, and *TRPM4* (rho = 0.977; p< 0.001).

### 3.2 Construction of co-expression modules

By setting the scale-free fit index at R^2^ = 0.80, a soft-thresholding power β = 14 was selected to best approximate the scale-free topology (**Figure S1A**). Genes were clustered based on 1-TOM dissimilarity measure, and the dynamic tree cut was set to 0.25 with minimal module size as 50 (**Figure S1B**). Seven distinct modules with module size ranging from 6,662 to 102 genes were identified (**Table S3**), and the green ME exhibited a significant correlation with tumour status (r = 0.5, p = 0.03; **Figure S1C**). MS comparison analysis also confirmed that the green module had the highest association with tumour status (**Figure S1D**).

### 3.3 Identification of hub genes

A positive correlation between GS and MM was identified, indicating that genes significantly associated with tumour status were also important representatives of the green module (correlation = 0.45, p = 1 × 10^-200^; **Figure S1E**). Moreover, GS and MM were significantly correlated with IMC (correlation = 0.49, p = 1 × 10^-200^ and correlation = 0.86, p = 1 × 10^-200^ for GS and MM, respectively; **Figures S1F, G**), which indicated that a gene with a high MM or GS also had a high intramodular connectivity and thus may represent hub genes in the module. Top 5% (~220) highly connected genes were screened from each cluster measure and the overlap analysis between the two clusters along with the absolute value of the MM >0.90 and GS >0.30, resulting in a total of 165 hub genes for further analysis (**Figure S1H**).

### 3.4 Pathways analysis

**Figure 2:**
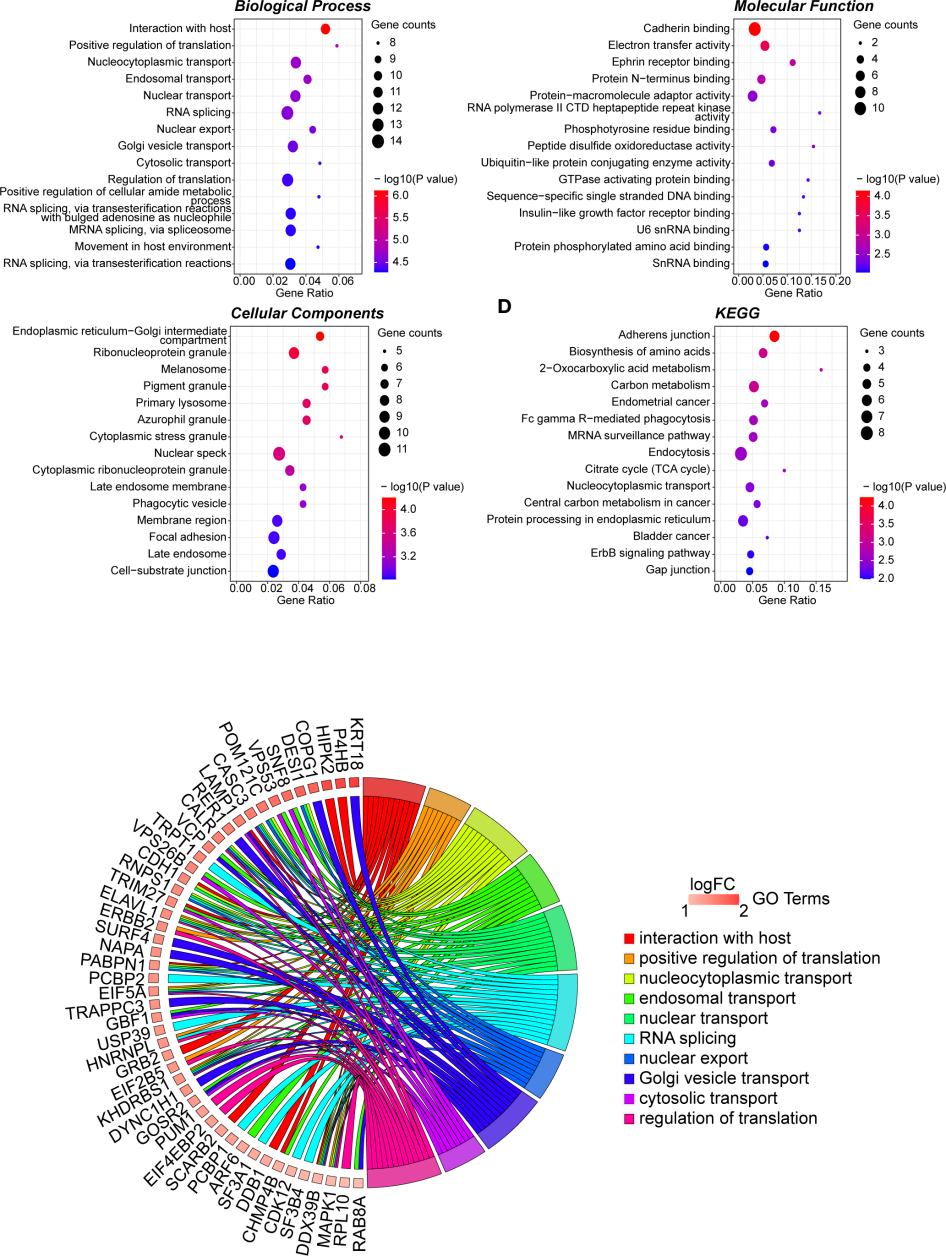
GO term analysis for differentially regulated genes. Dotplot for GO enrichment analysis in BP **(A)**, MF **(B)**, CC **(C)**, and KEGG pathway analysis **(D)** of the 165 hub genes in the green module. The larger the size of a dot, the greater enrichment of genes. The dot colour represents the -log10(p-value). **(E)** Network chord plot between the BP-enriched GO terms and their corresponding hub genes. The top 10 terms are presented, and the redder the box below the gene, the greater the log fold change. GO, Gene Ontology; KEGG, Kyoto Encyclopedia of Genes and Genomes; BP, Biological Process; MF, Molecular Function; CC, Cellular Component.

The 165 hub genes were enriched in “interaction with host,” “nucleocytoplasmic transport,” “nuclear transport,” “RNA splicing,” and “regulation of translation” GO biological processes (**Figure 2A** **;** **Table S4**). 'cadherin binding' was the most significantly enriched pathway in molecular function (MF) (**Figure 2B** **;** **Table S5**). The cellular component (CC) pathways were enriched in “nuclear speckle,” “focal adhesion,” “cell-substrate junction,” and “ribonucleoprotein granule” pathways (**Figure 2C** **;** **Table S6**). KEGG pathway analysis indicated that the hub genes participated in pathways including “adherens junction,” “biosynthesis of amino acids,” and “carbon metabolism” (**Figure 2D** **;** **Table S7**). The top 10 biological process GO terms and their assigned genes are shown in **Figure 2E**.

### 3.5 Protein–protein interaction network and hub gene identification

**Figure 3:**
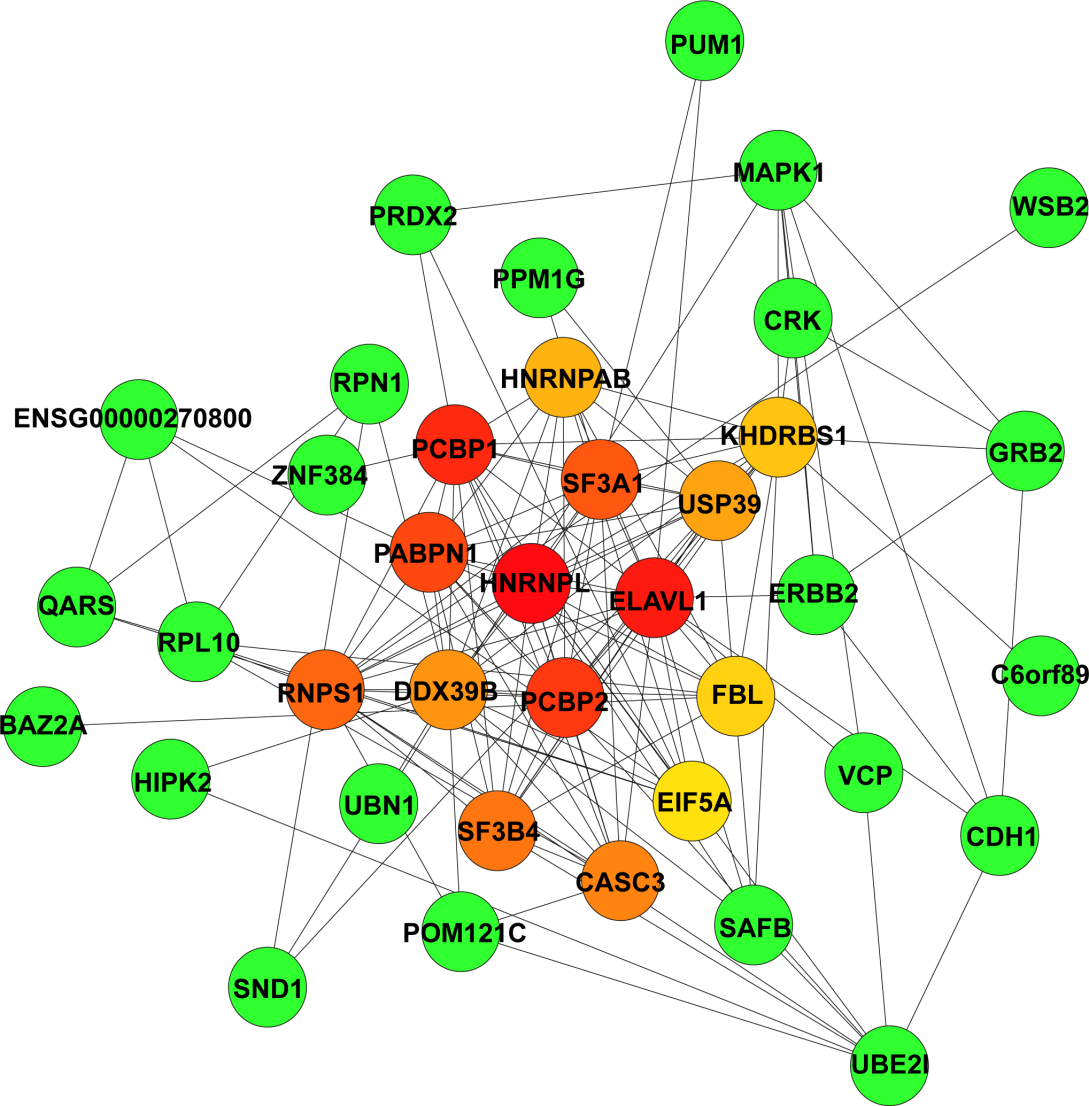
Identification of highly connected hub genes in the PPI network. PPI networks derived from Cytoscape using the cytoHubba plugin and the MCC algorithm for the 165 hub genes from the green module. Among the top 15 genes, the higher the MCC-based connectivity score is, the redder the colour of the node (gene). Neighbour genes in the module are presented in green. PPI, protein–protein interaction; MCC, maximal clique centrality.

The STRING database was used to construct the PPI network of the 165 hub genes in the green module. The resulting networks were further analysed and visualised in Cytoscape using the plugin CytoHubba, with the MCC algorithm for scoring gene connectivity (**Table S8**). The network of the top 15 genes and their neighbour genes is presented in **Figure 3**. The top 5 hub genes are *HNRNPL*, *ELAVL1*, *PCBP1*, *PCBP2*, and *PABPN1*, and notably that all of these genes were significantly enriched in the “RNA splicing” biological process (**Figure 2E**). For further analysis, the top 5 hub genes and the five identified DEGs were considered candidate genes.

### 3.6 Validation of candidate gene expression

The expression levels of the candidate genes (*HNRNPL*, *ELAVL1*, *PCBP1*, *PCBP2*, *PABPN1*, *PTPRF*, *MRPL24*, *DANCR*, *MYC*, and *TRPM4*) were validated between tumour and normal tissue samples in TCGA-PRAD dataset. These genes were defined as candidates on the basis that they were identified as “hub” genes in either the PPI network deriving from the most significant WGCNA module or represented the minimal differential expression signature required to distinguish tumour from benign tissues in the expression dataset. All genes, except for *PTPRF*, exhibited significantly altered expression between tumour and normal samples, with eight showing elevated expression {*HNRNPL* [p< 0.0001 and log2(FC) = 0.232], *ELAVL1* [p< 0.0001, log2(FC) = 0.179], *PCBP1* [p< 0.01, log2(FC) = -0.219], *PCBP2* [p< 0.001, log2(FC) = 0.171], *PABPN1* [p< 0.0001, log2(FC) = 0.529], *MRPL24* [p< 0.0001, log2(FC) = 0.432], *DANCR* [p< 0.0001, log2(FC) = 0.859], *MYC* [p< 0.0001, log2(FC) = 0.840], and *TRPM4* [p< 0.0001, log2(FC) = 1.738]; **Figure S2**}.

### 3.7 Construction of a five-gene signature for predicting prognosis in prostate cancer patients

**Figure 4:**
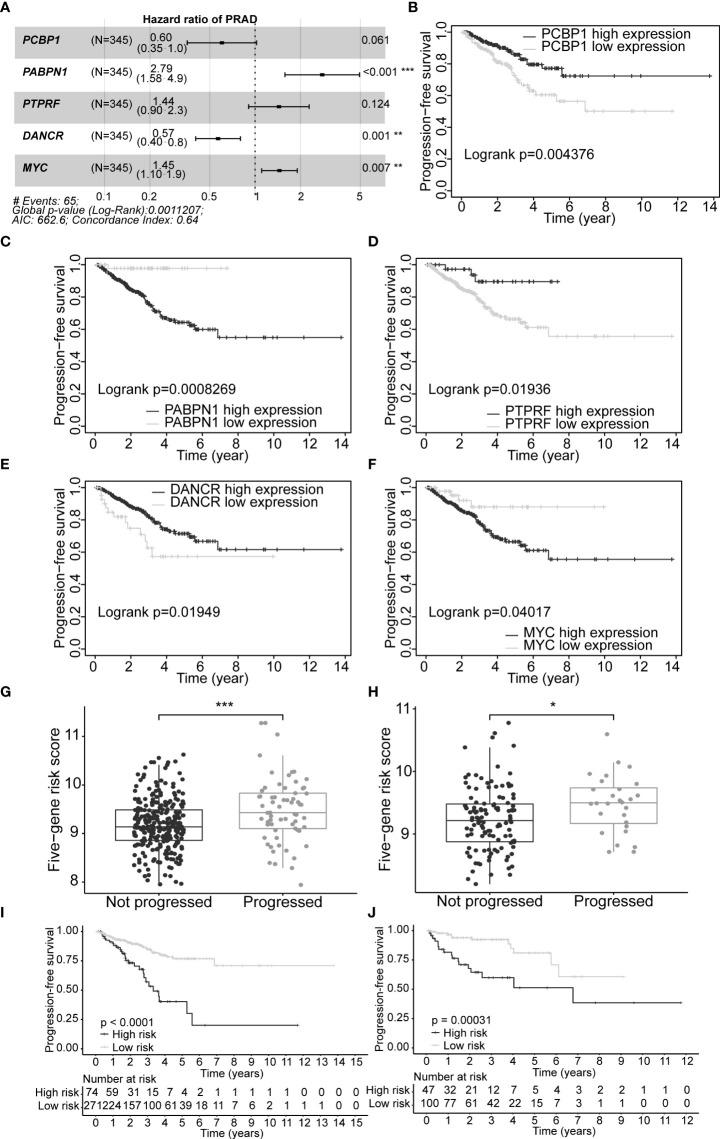
Construction of optimal gene signature and performance of the PFS survival risk score model. **(A)** Forest plot of the five genes in the signature. **(B–F)** KM survival curves and log-rank test based on gene expression levels for the 5 genes in the signature. Boxplots of the signature risk score between not progressed and progressed PCa patient groups in TCGA-PRAD training set **(G)** and TCGA-PRAD testing set **(H)**. The significance of the risk score difference between the two groups is interpreted as asterisks (ns, no significance, *p< 0.05, **p< 0.01, ***p< 0.001). KM survival analysis and log-rank test based on the risk scores obtained in TCGA-PRAD training set **(I)** and TCGA-PRAD testing set **(J)**. PFS, progression-free survival; KM, Kaplan–Meier; PCa, prostate cancer; TCGA, The Cancer Genome Atlas; PRAD, prostate adenocarcinoma.

A set of five genes (*PCBP1*, *PABPN1*, *PTPRF*, *DANCR*, and *MYC*) was retained after applying LASSO (Lambda minimum = 0.009302712; **Figures S3A, B**) and multivariate Cox regression analyses to the training cohort and was considered as an optimal prognostic model for PFS of PCa patients (**Table S9**, **Figure 4A**). In KM analysis, all five genes had significant differences between high- and low-expression groups [*PCBP1* (p = 0.004), *PABPN1* (p< 0.001), *PTPRF* (p = 0.019), *DANCR* (p = 0.019), and *MYC* (p = 0.040); **Table S10**]. Patients with high *PABPN1* and *MYC* gene expression had reduced PFS, whereas those with elevated *PCBP1*, *PTPRF*, and *DANCR* expression demonstrated improved PFS (**Figures 4B–F**). The predictive risk score was calculated as follows: survival risk score = (-0.508*PCBP1) + (1.026*PABPN1) + (0.363*PTPRF) + (-0.567*DANCR) + (0.372*MYC).

### 3.8 Evaluation of the model risk score

**Figure 5:**
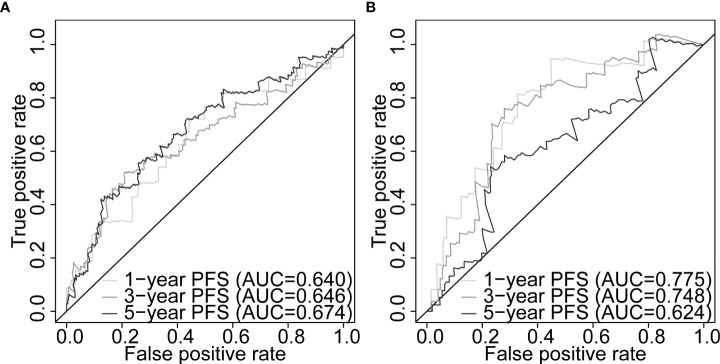
Five-gene signature evaluation of PFS in PCa patients. Time-dependent ROC curves assessing the five-gene signature performance for 1-, 3-, and 5-year PFS in TCGA-PRAD training set **(A)** and TCGA-PRAD testing set **(B)**. PCa, prostate cancer; PFS, progression-free survival; TCGA, The Cancer Genome Atlas; PRAD, prostate adenocarcinoma; ROC, receiver operating characteristic.

Our five-gene risk model showed a higher risk score in the disease progression patient group compared to that of the progression-free group in the training set (p< 0.001; **Figure 4G**) and the testing set (p< 0.05; **Figure 4H**). In both cohorts, the optimal separation value categorised patients ranked with ascending risk scores into high- and low-risk groups, and the number of progressed patients increased as the risk score increased (**Figures S4A, B**). Moreover, the low-risk patients showed better PFS than the high-risk patients (p< 0.0001 and p = 0.00031 for the training and testing set, respectively; **Figures 4I, J** **)**. Differential expression of our signature genes between the high- and low-risk groups revealed that all five genes were differentially expressed in the training set {*PABPN1* [p< 0.0001 and log2(FC) = 0.505], *MYC* [p< 0.01 and log2(FC) = 0.352], *PCBP1* [p< 0.0001 and log2(FC) = -0.341], *PTPRF* [p< 0.05 and log2(FC) = -0.159], and *DANCR* [p< 0.001 and log2(FC) = -0.412]}, whereas only three genes showed significant dysregulation in the testing set {*PABPN1* [p< 0.0001 and log2(FC) = 0.468], *MYC* [p< 0.05 and log2(FC) = 0.391], and *PCBP1* [p< 0.01 and log2(FC) = -0.239]} (**Figure S6A**). Other clinicopathological subgroups were also evaluated in a similar way (**Figures S6B–E**). Time-dependent ROC AUCs of 0.640, 0.646, and 0.674 (training set; **Figure 5A**) and 0.775, 0.748, and 0.624 (testing set; **Figure 5B**) were determined for 1-, 3-, and 5-year PFS. The C-index in the training set was 0.636 [95% confidence interval (CI): 0.554–0.717] and 0.709 (95% CI: 0.616–0.802) in the testing set. The calibration plots showed a high concordance between the predicted and actual outcomes of 1-, 3-, and 5-year PFS in TCGA-PRAD training set (**Figure S5A**), and 1- and 3-year PFS in TCGA-PRAD testing set (**Figure S5B**).

### 3.9 Prognostic ability of the signature with other clinicopathological factors

**Figure 6:**
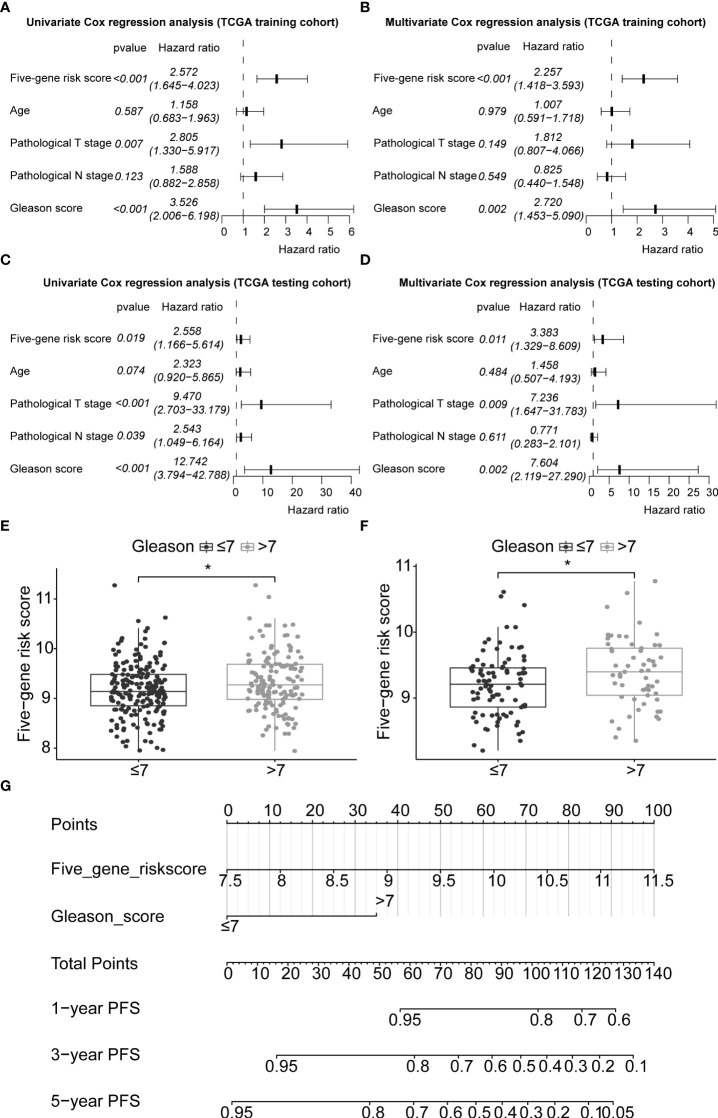
Application of the five-gene signature and nomogram construction to clinical practice. Univariate and multivariate Cox regression analyses of the five-gene signature risk score, age of the PCa patients at diagnosis, pathological T stage (tumour stage), pathological N stage (Lymph node status), and Gleason score in TCGA-PRAD training set **(A, B)** and TCGA-PRAD testing set **(C, D)**. Boxplots based on risk scores between Gleason scores ≤7 and >7 groups in TCGA-PRAD training set **(E)** and TCGA-PRAD testing set **(F)**. The significance of the risk score difference between the two groups is interpreted as asterisks (*p< 0.05). **(G)** Development of a nomogram to predict 1-, 3-, and 5-year PFS in the training set. PCa, prostate cancer; PFS, progression-free survival; KM, Kaplan–Meier; TCGA, The Cancer Genome Atlas; PRAD, prostate adenocarcinoma.

In this study, 291 and 125 patients remained after the exclusion of incomplete clinicopathological records from the training and testing sets, respectively. Our signature was significantly associated with PFS in both TCGA-PRAD sets [p< 0.001 and p = 0.0191, hazard ratios (HRs) = 2.57 (95% CI: 1.64–4.02) and 2.56 (95% CI: 1.17–5.61), respectively; **Tables 3**, **4**; **Figures 6A, C**]. The multivariate Cox regression analysis, after adjusting for covariate factors, demonstrated that the signature remained significant in both cohorts [p< 0.001 and p = 0.0106, HRs = 2.26 (95% CI: 1.42–3.59) and 3.38 (95% CI: 1.33–8.61), respectively; **Tables 3**, **4**; **Figures 6B, D**], suggesting that our signature may be used as an independent prognostic factor to predict PFS. The risk score of our signature demonstrated a significantly higher risk in the Gleason group >7 (p< 0.05 in both sets; **Figures 6E, F** **)**. Moreover, the risk score of our signature could significantly distinguish high- and low-risk patient groups of progression within the subgroups of all selected clinicopathological factors [i.e., Gleason score (≤7 *vs*. >7), pathological T stage (T2 *vs*. T3–T4), and pathological N stage (N0 *vs*. N1) ] in the training set. The results revealed that high-risk patients were associated with worse PFS rate than low-risk patients (p< 0.05; **Figures S7A–F** Left panel). Similar results were obtained from the testing set (p< 0.05; **Figures S7A–F** Right panel), except for the Gleason score >7 subgroup (p = 0.059; **Figure S7B** Right panel) and N1 stage subgroup (p = 0.06; **Figure S7F** Right panel).

### 3.10 Nomogram construction and prognostic model comparison

**Figure 7:**
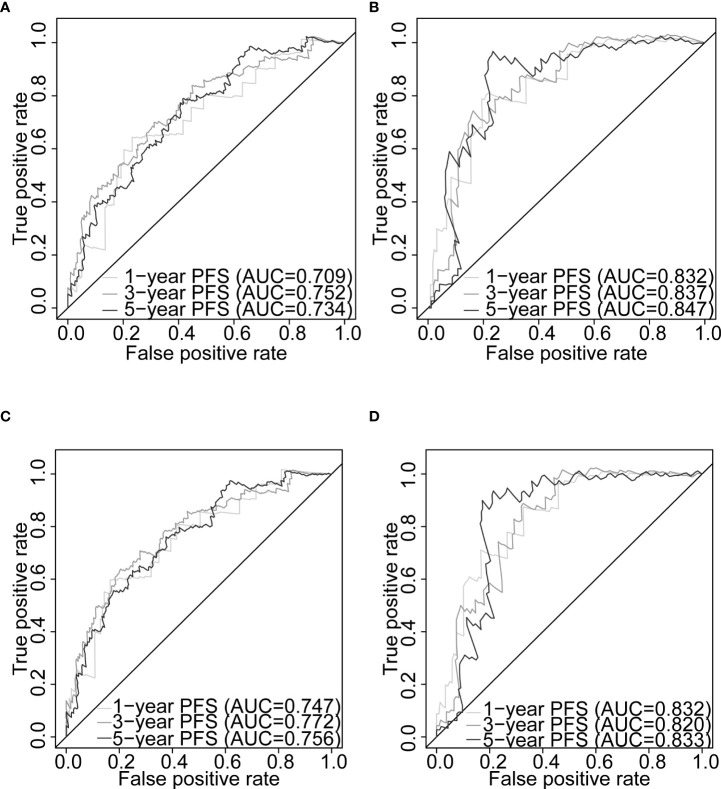
Nomogram evaluation of PFS in PCa patients. Time-dependent ROC curves assessing the nomogram model performance for 1-, 3-, and 5-year PFS, with the Gleason score classified into two subgroups (≤7 and >7) or five subgroups (6, 7, 8, 9, and 10) in TCGA-PRAD training set (**A**, **C**, respectively) and TCGA-PRAD testing set (**B**, **D**, respectively). PCa, prostate cancer; PFS, progression-free survival; TCGA, The Cancer Genome Atlas; PRAD, prostate adenocarcinoma; ROC, receiver operating characteristic.

Gleason score was predictively associated with PFS of PCa patients from both univariate and multivariate Cox regression analyses in the training set [p< 0.001 and 0.00176, HRs = 3.53 (95% CI: 2.01–6.20) and 2.72 (95% CI: 1.45–5.09), respectively; **Table 3**; **Figures 6A, B**] and the testing set [p< 0.001 and 0.00186, HRs = 12.7 (95% CI: 3.79–42.8) and 7.60 (95% CI: 2.12–27.3), respectively; **Table 4**; **Figures 6C, D**]. A nomogram consisting of the signature risk score and Gleason score was built to predict a PCa patient’s 1-, 3-, and 5-year PFS from the training set (**Figure 6G**). The ROC AUCs for predicting 1-, 3-, and 5-year PFS from the nomogram were 0.709, 0.752, and 0.734, respectively, in the training set (**Figure 7A**) and 0.832, 0.837, and 0.847, respectively, in the testing set (**Figure 7B**). The C-index of the nomogram was 0.701 (95% CI: 0.630–0.772) in the training set and 0.803 (95% CI: 0.732–0.875) in the testing set. The calibration plots of the nomogram also showed a satisfactory agreement between the predicted and actual observed probabilities of 1-, 3-, and 5-year PFS in the training (**Figure S8A**) and the testing (**Figure S8B**) sets. This indicated that the Gleason score enhanced our gene signature performance. Additionally, having categorised the Gleason score into five subgroups (i.e., 6, 7, 8, 9, and 10), the AUCs of the nomogram had a slight improvement compared to the nomogram that contains the Gleason score of two subgroups (i.e., ≤7 and >7) in the training cohort (AUCs: 0.747 *vs*. 0.709, 0.772 *vs*. 0.752, and 0.756 *vs*. 0.734; **Figures 7A, C** **)**, while the opposite was observed in the testing cohort (AUCs: 0.832 *vs*. 0.832, 0.820 *vs*. 0.837, and 0.833 *vs*. 0.847; **Figures 7B, D** **)** for predicting the three time points.

**Table 3:** Univariate and multivariate analyses of the five-gene-based risk score and clinicopathological characteristics in TCGA-PRAD training set (n = 291).

Variables	Univariate analysis				Multivariate analysis			
	HR	HR.95L	HR.95H	p-value	HR	HR.95L	HR.95H	p-value
**TCGA-PRAD training set (n = 291)**								
Five-gene risk score	2.57	1.64	4.02	<0.0001	2.26	1.42	3.59	<0.001
Age (<60 *vs*. ≥60 years)	1.16	0.68	1.96	0.59	1.01	0.59	1.72	0.98
Pathological T stage (T2 *vs*. T3–T4)	2.81	1.33	5.92	0.01	1.81	0.81	4.07	0.15
Pathological N stage (N0 *vs*. N1)	1.59	0.88	2.86	0.12	0.82	0.44	1.55	0.55
Gleason score (≤7 *vs*. >7)	3.53	2.01	6.20	<0.0001	2.72	1.45	5.09	0.002

TCGA, The Cancer Genome Atlas; PRAD, prostate adenocarcinoma; Age, age at diagnosis; T stage, tumour stage; N stage, lymph node status (N0 = without lymph node metastasis; N1 = with lymph node metastasis); HR, hazard ratio; HR.95L/H, 95 confidence interval of hazard ratio lower/upper bound.

**Table 4:** Univariate and multivariate analyses of the five-gene-based risk score and clinicopathological characteristics in TCGA-PRAD testing set (n = 125).

Variables	Univariate analysis				Multivariate analysis			
	HR	HR.95L	HR.95H	p-value	HR	HR.95L	HR.95H	p-value
**TCGA-PRAD testing set (n = 125)**								
Five-gene risk score	2.59	1.17	5.617	0.02	3.38	1.33	8.61	0.01
Age (<60 *vs*. ≥60 years)	2.32	0.92	5.87	0.07	1.46	0.51	4.19	0.48
Pathological T stage (T2 *vs*. T3–T4)	9.47	2.70	33.2	<0.001	7.24	1.65	31.8	0.01
Pathological N stage (N0 *vs*. N1)	2.54	1.05	6.16	0.04	0.77	0.28	2.10	0.61
Gleason score (≤7 *vs*. >7)	12.7	3.79	42.8	<0.0001	7.60	2.12	27.3	0.001

TCGA, The Cancer Genome Atlas; PRAD, prostate adenocarcinoma; Age, age at diagnosis; T stage, tumour stage; N stage, lymph node status (N0 = without lymph node metastasis; N1 = with lymph node metastasis); HR, hazard ratio; HR.95L/H, 95 confidence interval of hazard ratio lower/upper bound.

The CPG scores of the PCa patients were calculated for both the training set (**Table S11**) and the testing set (**Table S12**). In this study, 304 patients (training set) and 127 patients (testing set) were included to compare our gene signature and nomogram with the CPG risk stratification model. The ROC analysis demonstrated that our nomogram outperformed the CPG model for 1-, 3-, and 5-year PFS predictions in the training set (AUCs: 0.755 *vs*. 0.673; 0.749 *vs*. 0.683; 0.728 *vs*. 0.650; **Figures S9A–C**) and 1- and 3-year PFS predictions in the testing set (AUCs: 0.846 *vs*. 0.823; 0.846 *vs*. 0.790; **Figures S9D, E**). In the training set, our nomogram alone predicted better than when it was combined with the CPG score (AUCs: 0.755 *vs*. 0.737; 0.749 *vs*. 0.740; 0.728 *vs*. 0.701; **Figures S9A–C**). The predictive power of the joint gene signature and the CPG model was superior to the CPG alone for 1-, 3-, and 5-year PFS in the training set (AUCs: 0.733 *vs*. 0.673; 0.733 *vs*. 0.683; 0.727 *vs*. 0.650; **Figures S9A–C**) and the testing set (AUCs: 0.859 *vs*. 0.823; 0.859 *vs*. 0.790; 0.901 *vs*. 0.889; **Figures S9D–F**). For the prediction of 1-, 3-, and 5-year PFS in the training set, the gene signature had higher AUCs than the T stage and PSA level (0.666 *vs*. 0.578 *vs*. 0.575, 0.664 *vs*. 0.609 *vs*. 0.570, 0.691 *vs*. 0.603 *vs*. 0.548, respectively; **Figures S9A–C**). In both cohorts, we found that the nomogram outperformed the conventional clinical parameters that defined the CPG model, including Gleason score, pathological T stage, and PSA level, for predicting PFS at 1-, 3-, and 5-year intervals (training set: AUCs: 0.755 *vs*. 0.680 *vs*. 0.578 *vs*. 0.575; 0.749 *vs*. 0.685 *vs*. 0.609 *vs*. 0.570; 0.728 *vs*. 0.641 *vs*. 0.603 *vs*. 0.548; **Figures S9A–C**; testing set: 0.846 *vs*. 0.770 *vs*. 0.754 *vs*. 0.474; 0.846 *vs*. 0.740 *vs*. 0.700 *vs*. 0.510; 0.876 *vs*. 0.826 *vs*. 0.799 *vs*. 0.495; **Figure S9D–F**).

## 4 Discussion

Prediction of the clinical course of disease remains a challenge in managing PCa. In this study, we undertook transcriptome-wide gene expression profiling of paired benign and malignant prostate samples obtained from radical prostatectomy of nine patients with histologically confirmed PCa. We carried out an integrated bioinformatics analysis to identify DEGs and modules from our self-generated Clariom D Human array data and constructed and validated a novel five-gene-based signature risk model based on the expression of the *PCBP1*, *PABPN1*, *PTPRF*, *DANCR*, and *MYC* genes for PFS using a publicly available TCGA-PRAD dataset. Our signature was able to stratify PCa patients into high- and low-risk groups based on the entire patient population and within some subgroups of the Gleason score, pathological T stage, and pathological N stage. Our gene signature also demonstrated good predictive power for PFS and remained as an independent prognostic factor from the multivariate Cox regression analysis after adjusting for clinicopathological factors including age, pathological tumour stages T and N, and Gleason score.

The combined signature and Gleason score nomogram gave a good predictive performance for 1-, 3-, and 5-year PFS in TCGA-PRAD training and testing sets and excellent calibration plots in both data sets, indicating a potential clinical application. Our nomogram consistently outperformed both the signature and the CPG model alone for predicting 1-, 3-, and 5-year PFS. The only exception is a 1.3% worse prediction of 5-year PFS in the testing set compared to the CPG model, which is a result of conducting the study exclusively on TCGA dataset. A higher prediction accuracy was also observed when the gene signature and CPG were combined compared to the CPG alone in both sets.

Four out of the five genes in our signature have all previously been implicated in malignancy. Poly(rC) binding protein 1 (*PCBP1)* has been demonstrated previously to be a PCa tumour suppressor gene (39). Loss of *PCBP1* can result in the upregulation of genes such as mitogen-activated protein kinase 1 (*MAPK1*) and extracellular signal-regulated kinase 2 (*ERK2*), which are overexpressed in PCa samples and known to be involved in tumorigenesis and metastatic progression (40). MYC is a well-established proto-oncogene that drives the tumorigenesis of PCa, and its activation was reported to be one of the first changes that occur just before or during the onset of prostate intraepithelial neoplasia (41). *MYC* is known to be expressed at high levels in the early stages of tumorigenesis, but also in more advanced PCa cells compared to normal or benign cells (42–44). Differentiation antagonizing non-protein-coding RNA (*DANCR*) is a long non-coding RNA that is known to enhance the invasion and metastasis of prostate cells by repressing TIMP2/3, crucial inhibitors of PCa metastasis (45). Downregulation of Protein tyrosine phosphatase, receptor type F (*PTPRF*) expression has been reported in advanced tumour stages and in poor OS prognosis in gastric cancer. Conversely, overexpression of *PTPRF* has been shown to suppress gastric tumour migration and invasion by deactivating ERK1/2 signalling (46). High expression of *PTPRF* has also been reported to reduce cell invasion, migration, and advanced metastasis potential in breast cancer (47). Poly(A)-binding protein nuclear 1 (*PABPN1*) is an RNA-processing gene. Although no role for linear *PABPN1* species in cancer has yet been reported, a circular RNA deriving from this locus has been reported to enhance colorectal cancer development by attenuating the activity of the splicing factor SRSF1 *via* the miR-638 axis (48).

GO enrichment analysis of the green module hub genes uncovered a number of enriched biological processes, including pathways linked to splicing processes and nucleocytoplasmic transport (**Figure 2A**, **Table S4**). All of our PFS signature genes had involvement in RNA splicing processes, and all of the 165 green module hub genes were implicated in regulatory splicing functions, processes, and pathways on the level of RNA. Aberrant alternative splicing (AS) is a major feature of cancer (49). Abnormal AS processes can activate and dysregulate the expression of oncogenes, as well as deactivating tumour suppressor genes, leading to cancer development and promotion of the progression of malignancies (50, 51). Previous studies demonstrated that abnormal AS machinery is strongly associated with the progression and aggressiveness of PCa (52–54) and can result in drug resistance in PCa cells [e.g., hormone resistance due to AS of androgen receptor (AR)] (51, 55). Disrupted nucleocytoplasmic transport processes have also been demonstrated to be associated with the development of many cancer types, including PCa (56).

A number of molecular biomarkers have been identified to predict different survival endpoints of the PCa patient, including OS, DFS, and RFS. For example, a previous study (9) established a Gleason score-related three-gene signature (*CDC45*, *ESPL1*, and *RAD54L*) to predict OS (AUC = 0.606, 0.562, and 0.608 for 1-, 3-, and 5-year OS in the GSE16560 dataset; AUC = 0.585, 0.552, and 0.495 for 1-, 3-, and 5-year OS in the GSE53922 dataset) and for DFS (AUC = 0.765, 0.698, and 0.628 for 1-, 3-, and 5-year DFS in the entire TCGA-PRAD dataset). *MYC* was overlapped between our signature and an autophagy-related gene signature (*FAM215A*, *FDD*, *MYC*, *RHEB*, and *ATG16L1*) for OS prediction (AUC = 0.84 in TCGA-PRAD dataset) from the study (57). Moreover, Gao et al. (58) proposed a signature consisting of six RBPs (*MSI1*, *LENG9*, *REXO2*, *PABPC1L*, *MBNL2*, and *RNASE1*) to predict 1-, 3-, and 5-year RFS in TCGA-PRAD cohort (AUCs: 0.799, 0.736, and 0.714) and the Memorial Sloan Kettering Cancer Center (MSKCC) cohort (AUCs: 0.693, 0.708, and 0.708). Meng et al. (59) identified 11 genes that were associated with RFS and developed them into a risk signature that could predict RFS over 1, 3, and 5 years in five datasets (TCGA-PRAD: 0.717, 0.711, and 0.641; MSKCC: 0.908, 0.898, and 0.857; GSE116918: 0.936, 0.735, and 0.705; GSE70768: 0.816, 0.706, and 0.554; GSE70769: 0.858, 0.806, and 0.745). Another study from Meng et al. (60) proposed a clinical and gene-based signature, including Gleason score, pathology T stage, *KLF5*, and *KLF13*, for the prediction of RFS over the same three time points. The predictive value of the model was evaluated in three cohorts (TCGA-PRAD: 0.735, 0.696, and 0.785; MSKCC: 0.854, 0.845, and 0.722; GSE116918: 0.832, 0.574, and 0.635).

Some progression-related signatures have been reported in advanced PCa. Previously, a 49 gene-based signature for prediction of metastatic-lethal (ML) progression had AUCs of 0.76, 0.77, and 0.83 for the signature alone, the clinical factors alone, and the combined models in the Fred Hutchinson (FH) dataset (11). A two-gene signature based on *KLK3* and *BIRC5* expression was evaluated for predicting circulating tumour cell (CTC) levels in metastatic castration-resistant PCa (mCRPC) that produced an AUC of 0.74 from a set of 29 mCRPC and 19 healthy individuals (12). A collection of signatures were selected and developed by a binary classifier, a support vector machine (SVM), in order to predict different pairs of PCa progression stages/substages (all AUCs >0.8) (13). In Hamzeh et al. (14) , a selection of signatures were derived using SVM and achieved high accuracy in their ability to predict three classes of tumour locations in PCa (left: *FBOX21*, *RTN1*, *NDUFA5*, and *POP7*; right: *ALG5*, *Z99129*, *SNAI2*, *MRI1*, and *MAF7*; bilateral: *HLA-DMB*, *SRSF6*, and *EIF4G2*; all AUCs = 0.99).

However, none of the above progression-related signatures were constructed using Cox regression (i.e., they were not suitable for PFS prediction), but rather a less preferred logistic regression or some other multi-level classification models, which do not account for survival time and censoring data (61). In light of this, we believe that our five-gene signature may be the very first model suitable for 1-, 3-, and 5-year prediction of the PFS, as well as its combined nomogram model.

A strength of our approach is the use of paired benign and malignant samples from the same patients. Gene expression data are frequently “noisy” and influenced by anthropometric and lifestyle traits that can be difficult to control for even in a multivariate fully adjusted model. The use of paired samples reduces noise; samples will have been exposed to the same confounding factors that gives us greater power than a standard case–control design. The limitations of our study relate to the restricted number of samples we were able to obtain for transcriptomic profiling (which has been partially addressed through replication in the publicly available TCGA-PRAD dataset) and the relatively small amount of clinical data available for each. These samples were collected as part of the routine clinical care pathway in line with tissue bank ethics, so we were restricted by data availability. However, although TCGA-PRAD dataset includes sufficient samples, it is predominantly based on prostate specimens from Caucasians (62), which may require extra attention when applying our gene signature to PCa patients from other ethnicities. Since our gene signature and nomogram were established and validated using TCGA database, additional transcriptome-wide datasets with a greater number of patients included and more clinical information (specifically the PFI and the progression status) are crucial for the external validation of our prognostic model. Moreover, although the CPG model is a well-validated prognostic tool for PCa, it was configured for predicting cancer-specific survival of non-metastatic PCa. In the future, it will be necessary to conduct further studies to compare available models including ours with other prognostic tools and assess the predictability of not only PFS but also other survival endpoints, such as OS.

In conclusion, we have carried out a transcriptome-wide expression profile in nine paired benign and malignant PCa samples and constructed a novel five-gene-based prognostic model (*PCBP1*, *PABPN1*, *PTPRF*, *DANCR*, and *MYC*) that is capable of classifying PCa patients into high- and low-risk groups with respect to PFS. Our gene signature has an effective potential to predict PFS of PCa patients who have undergone radical treatment and can be used as an independent prognostic factor from some traditional clinicopathological factors. Our nomogram alone resulted in an improved PFS prediction performance than traditional clinical features in PCa, including Gleason score, pathological T stage, and serum PSA levels. Moreover, both the nomogram and the gene signature combined with the CPG score model proved to be reliable prognostic models and performed better than the CPG model alone in predicting PCa PFS. Our signature may therefore have clinical utility as prognostic biomarkers, potentially predicting PFS in patients with PCa from small amounts of sample material, and facilitate decisions regarding treatment options for PCa patients. Future work should focus on validating our models in additional ethnically diverse sets, predicting the PFS in active surveillance PCa patient groups, and evaluating their clinical utility in accessible tissues, such as blood or urine.

## Data availability statement

The original contributions presented in the study are included in the article/**Supplementary Materials**. Further inquiries can be directed to the corresponding author.

## Ethics statement

The samples were obtained *via* the ethically approved Royal Devon and Exeter Tissue Bank (rec no: 16/SC/0162, with our project specific approval code- STB20). The patients/participants provided their written informed consent to participate in this study.

## Author contributions

ZM carried out the analysis and wrote the draft. JS contributed to statistical analysis and reviewed the draft. BK provided samples and clinical information and reviewed the draft. JJ advised on clinical interpretation and reviewed the draft. PM carried out the histological analysis and reviewed the draft. JM provided clinical interpretation and reviewed the draft. LH managed the project and reviewed the draft. All authors contributed to the article and approved the submitted version.

## Conflict of interest

LH is founder, director and chief scientific officer of SENISCA Ltd. SENISCA’s commercial activities have no bearing on the content of this publication.

The remaining authors declare that the research was conducted in the absence of any commercial or financial relationships that could be construed as a potential conflict of interest.

## Publisher’s note

All claims expressed in this article are solely those of the authors and do not necessarily represent those of their affiliated organizations, or those of the publisher, the editors and the reviewers. Any product that may be evaluated in this article, or claim that may be made by its manufacturer, is not guaranteed or endorsed by the publisher.
