# Supplementary material for: Gene expression analysis reveals a 5-gene signature for progression-free survival in prostate cancer
Source: Front Oncol. 2022 Aug 12;12:914078. doi: 10.3389/fonc.2022.914078 (PMC9413154; doi:10.3389/fonc.2022.914078)
Supplement: Supplementary file 1 [file DataSheet_1.docx]

Supplementary Material

# Supplementary Figures and Tables

## Supplementary Figures


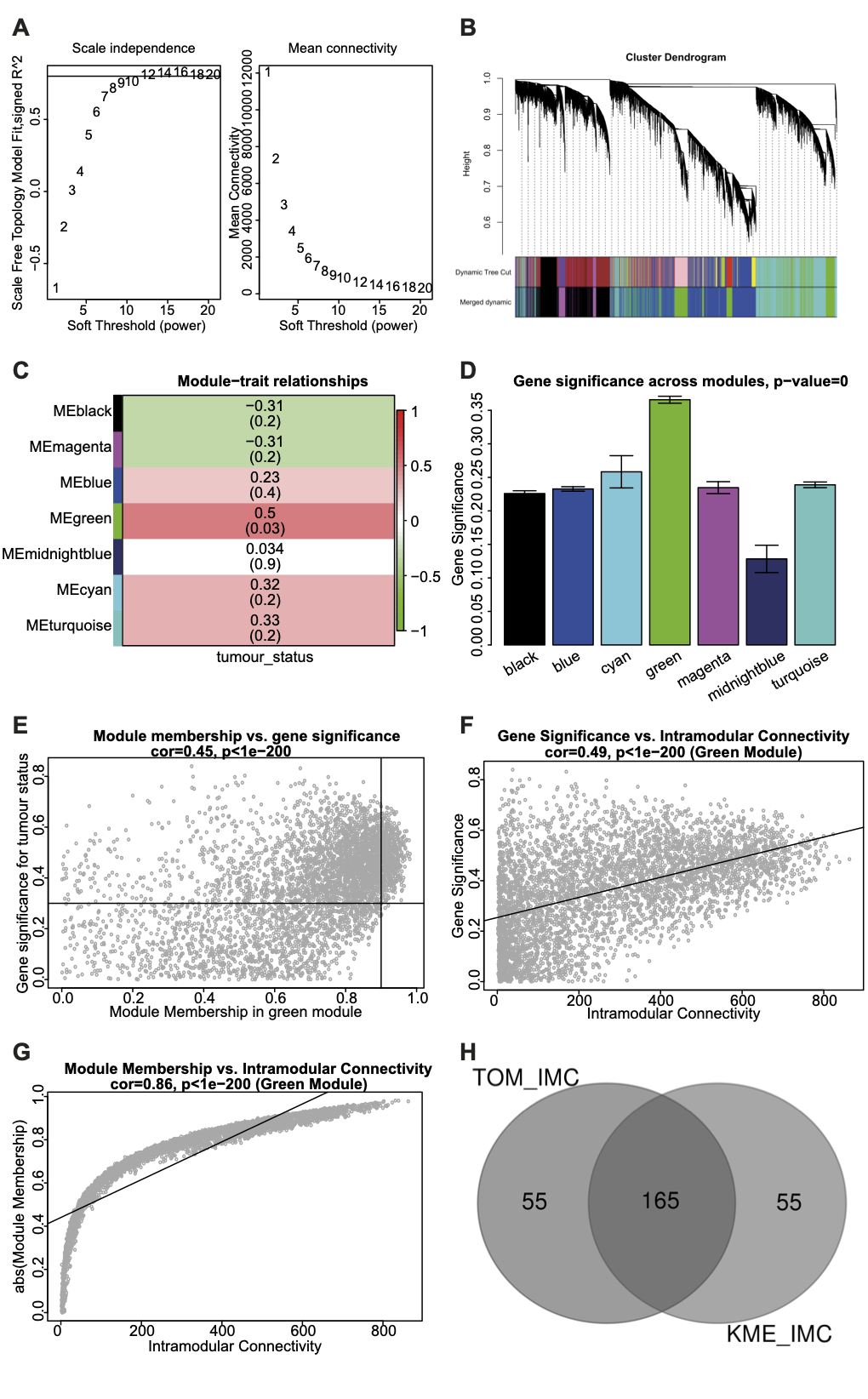


**Supplementary Figure 1.** WGCNA analysis and identification of hub genes associated with tumour status. **(A)** Analysis of network topology for various soft-thresholding powers and the determination of the power using the minimal R^2^ value 0.8 of the scale-free fit index (left) and the mean connectivity of the WGCNA (right). **(B)** Dendrogram of clustered genes and the corresponding modules. **(C)** Heatmap of Pearson correlation between tumour status and MEs. **(D)** A bar chart illustrating the module significance (mean gene significance) across genes in the corresponding modules. Screening for potential hub genes using correlation scatter plots in the green module: **(E)** GS vs. MM (genes in the top-right panel from the (black) crossed lines are highly correlated with tumour status), **(F)** GS vs. IMC, and **(G)** MM vs. IMC. The (black) crossed lines were set at MM=0.9 and GS=0.3; the (black) inclined lines indicate the line of best fit. **(H)** Venn diagram of overlapping genes between topological overlap matrix (TOM)- and module membership (KME)-based IMC measures, in the green module. WGCNA: weighted gene co-expression network analysis; ME: module eigengene; GS: Gene significance; MM: module membership; IMC: intramodular connectivity.

**
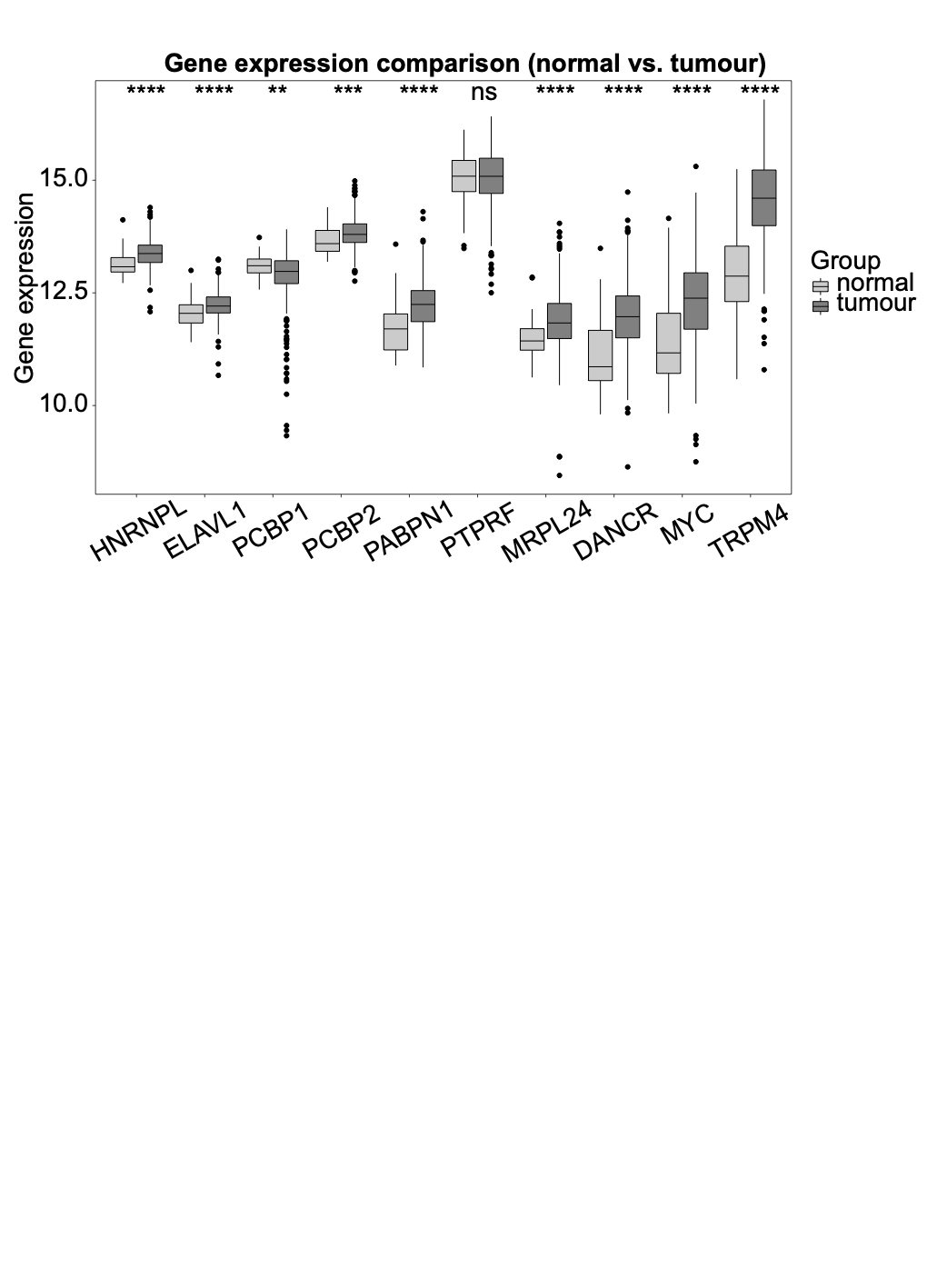
**

**Supplementary Figure 2.** Box plots of the expression levels of candidate genes between prostate sample status (normal vs. tumour) in the TCGA-PRAD dataset. The significance of differential gene expression between the two groups are interpreted as asterisks (ns: no significance, *P < 0.05, **P < 0.01, ***P < 0.001, ****P < 0.0001). TCGA: The Cancer Genome Atlas; PRAD: prostate adenocarcinoma.

**
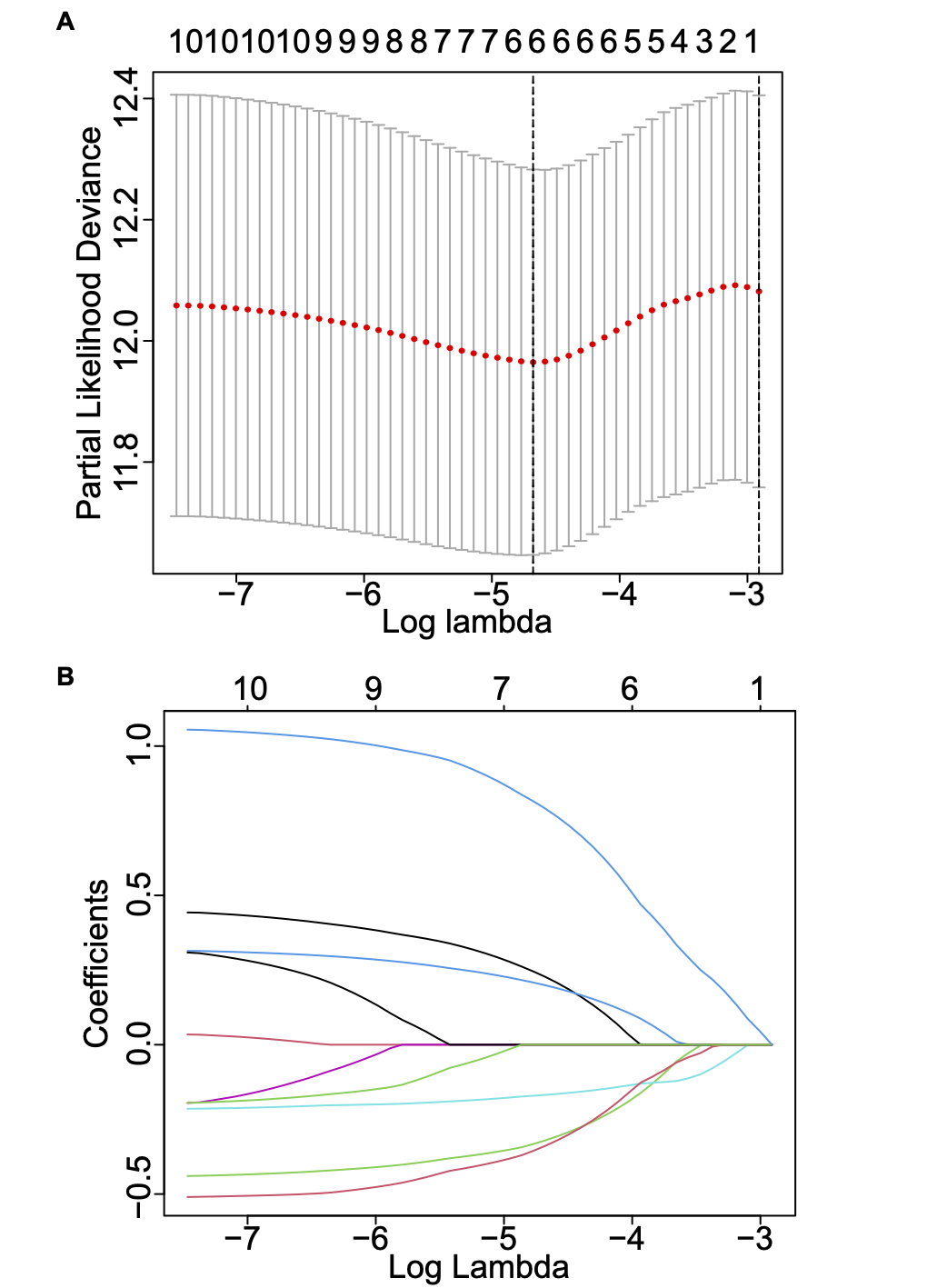
**

**Supplementary Figure 3.** Gene screening with LASSO regression analysis for PFS in PCa. **(A)** Selection of a suitable Lambda value. The red dots indicate the values of partial likelihood deviance. The black dashed lines show log (Lambda) values for Lambda minimum (left) and Lambda 1SE (right). Grey whiskers accompany the red dots and indicate ± SE. Ten-fold cross-validation was used to select the Lambdas. **(B)** Coefficient profiles for all input genes. PCa: prostate cancer; PFS: progression-free survival; LASSO: least absolute shrinkage and selection operator; SE: standard error.

**
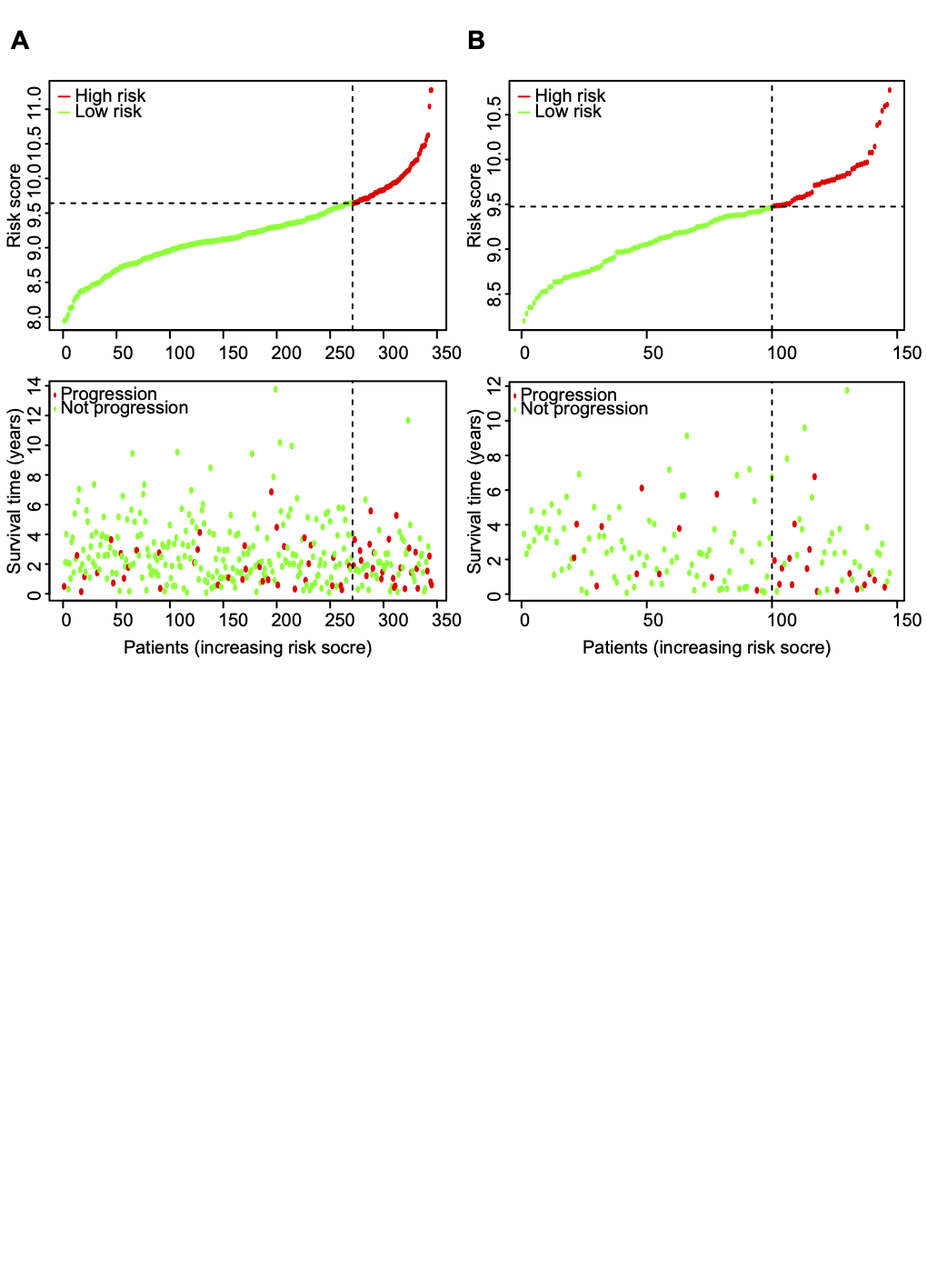
**

**Supplementary Figure 4.** An illustration of the risk score distribution (upper panel) and the progression classification of the PCa patients (progressed or not progressed, lower panel) in the TCGA-PRAD training cohort **(A)** and the TCGA-PRAD testing cohort **(B)**. TCGA: The Cancer Genome Atlas; PRAD: prostate adenocarcinoma.

**
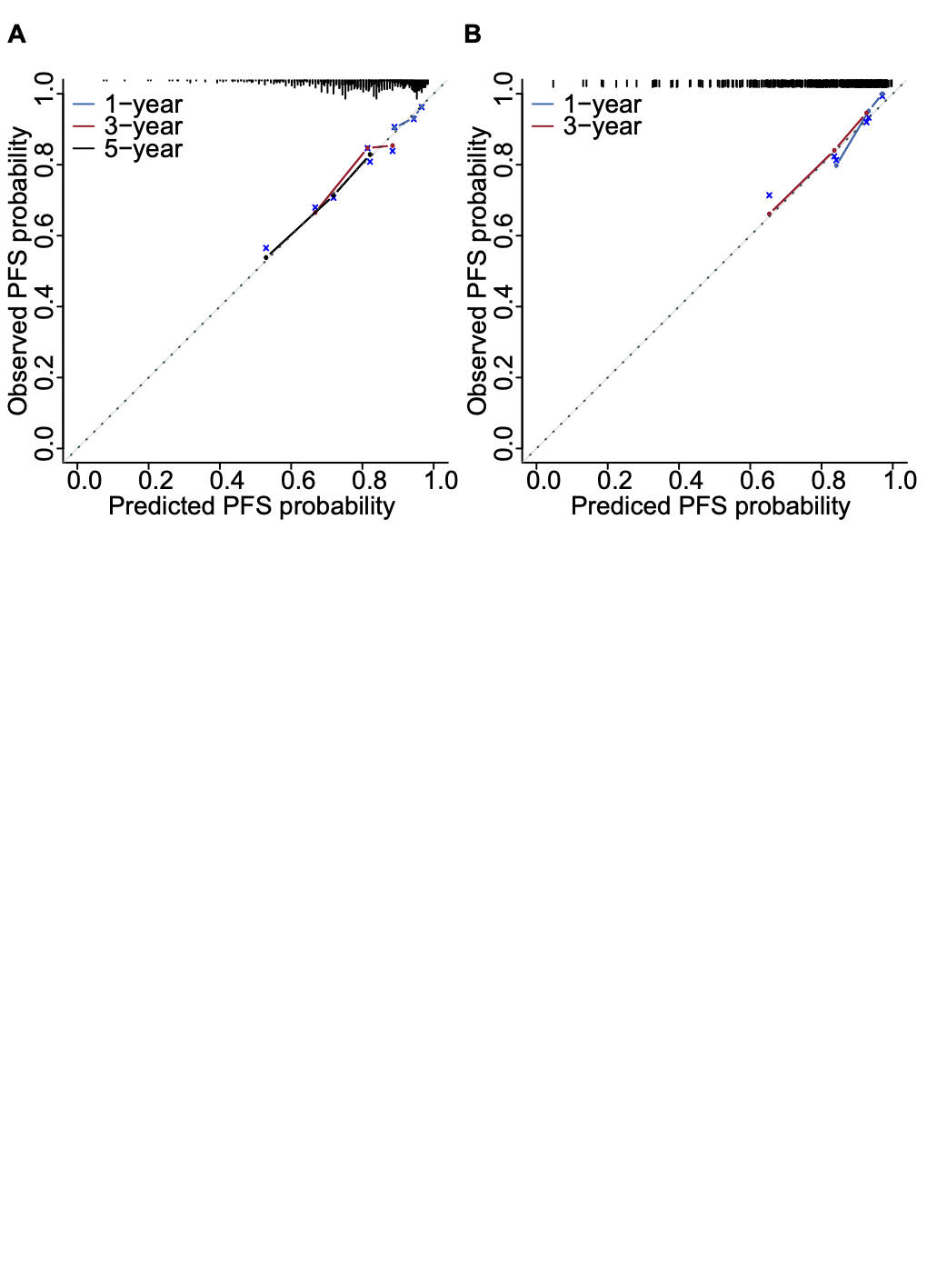
**

**Supplementary Figure 5.** Five-gene signature model evaluation for predicting PFS of PCa patients. Calibration plots of PFS probability prediction accuracy for 1-, 3-, and 5-year in the TCGA- PRAD training cohort **(A)** and for 1- and 3-year in the TCGA-PRAD testing cohort **(B)**. PCa: prostate cancer; PFS: progression-free survival; TCGA: The Cancer Genome Atlas; PRAD: prostate adenocarcinoma; ROC: Receiver operating characteristic.

**
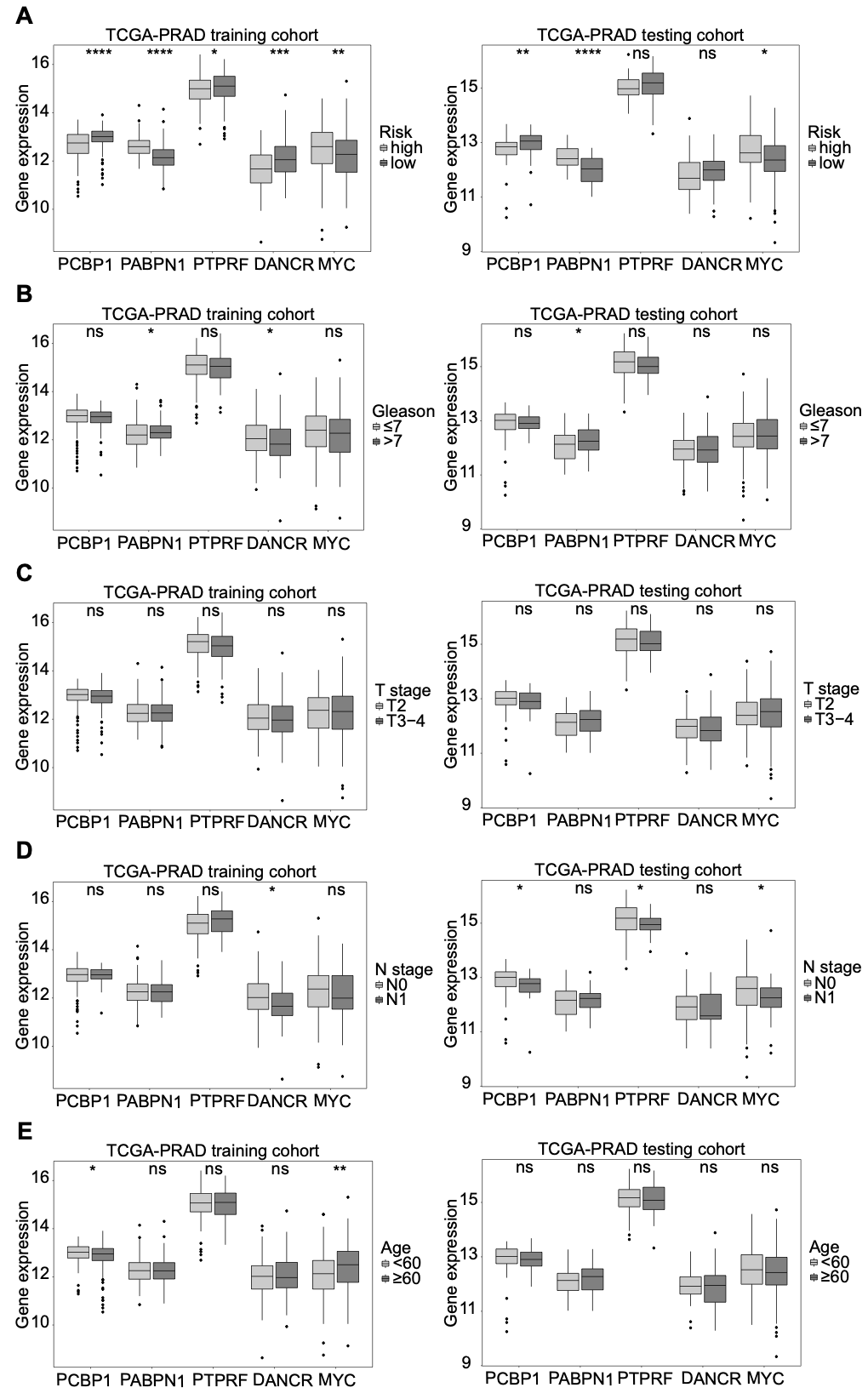
**

**Supplementary Figure 6.** Boxplots of differential gene expression of 5 genes in the risk model between different risk groups **(A)**, different Gleason score groups **(B)**, different pathological T stage groups **(C)**, different pathological N stage groups **(D)**, and different age groups **(E)** in the TCGA- PRAD training cohort (left panel) and the TCGA-PRAD testing cohort (right panel), respectively. The significance of differential gene expression between the two groups are interpreted as asterisks (ns: no significance, *P < 0.05, **P < 0.01, ***P < 0.001, ****P < 0.0001). TCGA: The Cancer Genome Atlas; PRAD: prostate adenocarcinoma.

**
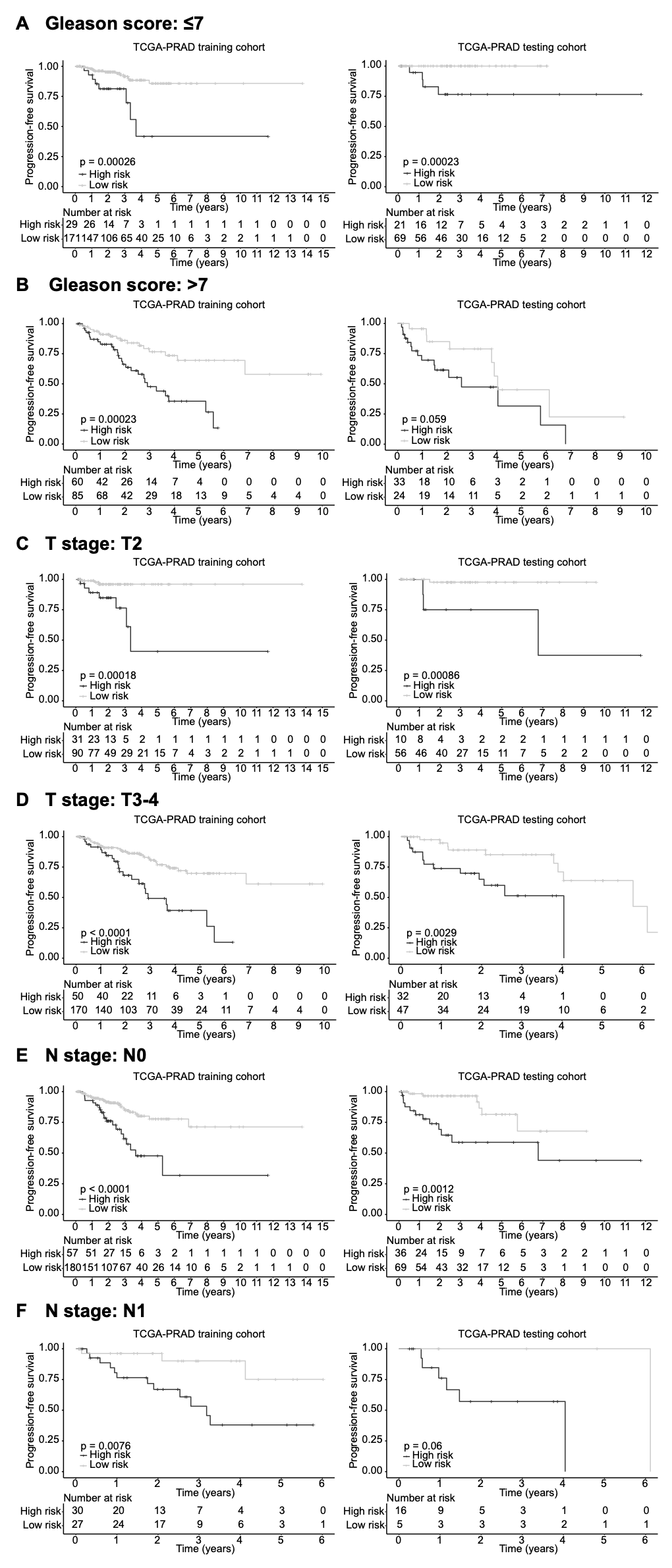
**

**Supplementary Figure 7.** KM survival curves of PFS according to the risk score in patients stratified by Gleason score ≤ 7 **(A)**, >7 **(B)**; pathological T stage T2 **(C)**, T3-4 **(D)**; and pathological N stage N0 **(E)**, N1 **(F)** in the TCGA-PRAD training cohort (left panel) and the TCGA-PRAD testing cohort (right panel), respectively. PFS: progression-free survival; KM: Kaplan-Meier; TCGA: The Cancer Genome Atlas; PRAD: prostate adenocarcinoma.

**
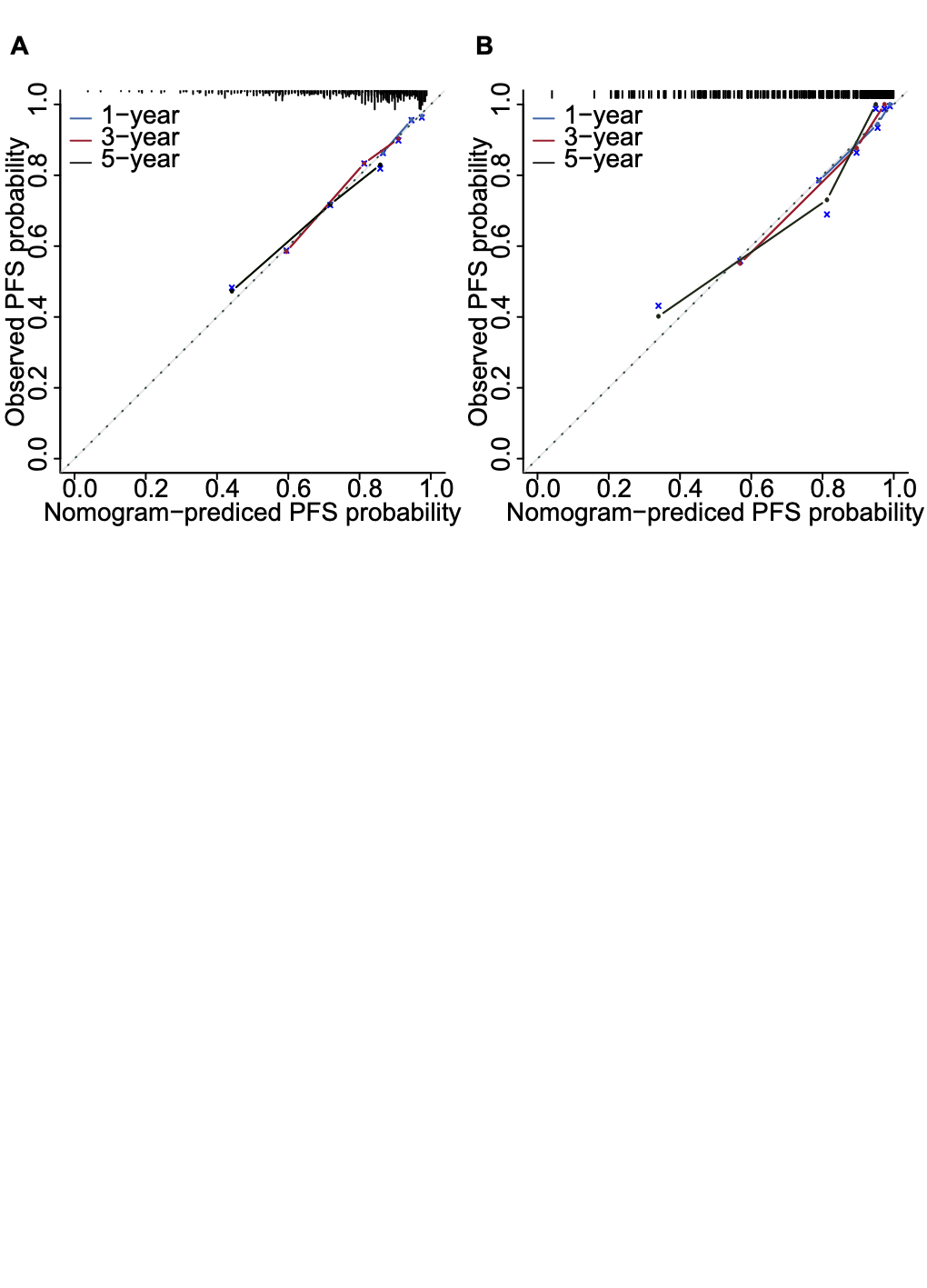
**

**Supplementary Figure 8.** Nomogram evaluation of PFS in PCa patients. Calibration plots of PFS probability prediction accuracy for 1-, 3-, and 5-year in the TCGA-PRAD training cohort **(A)** and the TCGA-PRAD testing cohort **(B)**. PCa: prostate cancer; PFS: progression-free survival; TCGA: The Cancer Genome Atlas; PRAD: prostate adenocarcinoma; ROC: Receiver operating characteristic.

**
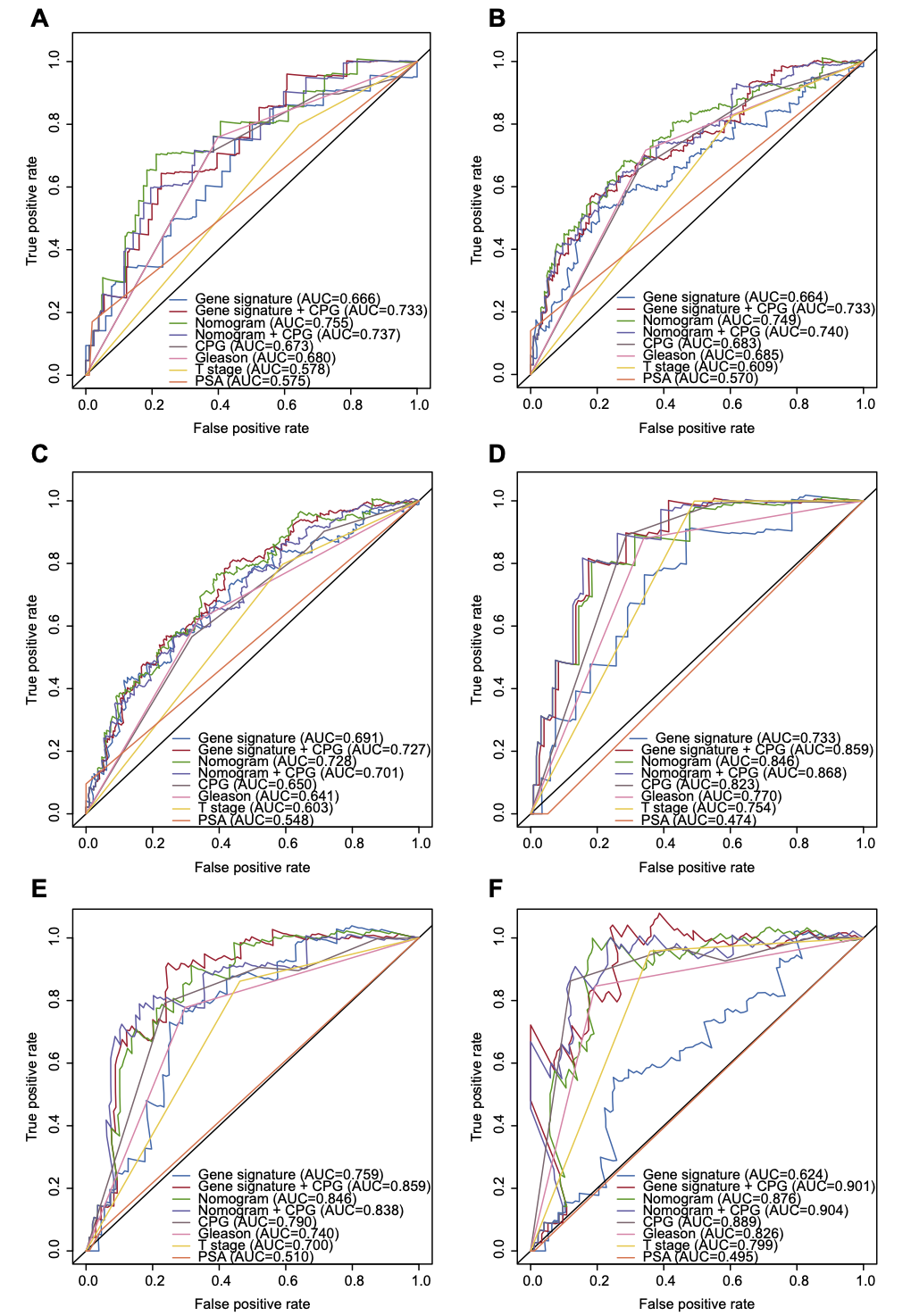
**

**Supplementary Figure 9.** Evaluation of prognostic models of PFS in PCa patients. The time-dependent ROC curves assessing the performance of the five-gene signature, the gene signature plus the CPG score, the nomogram, the nomogram plus the CPG score, the CPG score, the Gleason score, the pathological T stage, and the PSA level, for 1-, 3-, and 5-year PFS in the TCGA-PRAD training cohort (**A-C**, respectively) and the TCGA-PRAD testing cohort (**D-F**, respectively). PCa: prostate cancer; PFS: progression-free survival; CPG: Cambridge Prognostic Groups; PSA: prostate-speciﬁc antigen; TCGA: The Cancer Genome Atlas; PRAD: prostate adenocarcinoma; ROC: Receiver operating characteristic.

## Supplementary Tables

**Supplementary Table 1.** Differentially expressed gene (DEG) list. logFC: log2 transformed fold change; AveExpr: average expression (log2-transformed) for the gene over all samples; t: moderared t-statistic; P.Value: raw p-value; adj.P.Val:adjusted p-value using Benjamini-Hochberg (BH) approach; B: log-odds; change: down- or up-regulated gene.

**Supplementary Table 2.** BIOENV analysis to identify the DEG subsets. Identification of the most representative subsets of DEGs that encapsulate the information in the complete DEG set. Spearman rank correlation method was used and top 5 selections of genes are shown on the table. All these 5 subset selections are strongly correlated with the full DEGs data set. DEG: differentially expressed gene.

**Supplementary Table 3.** WGCNA modules and their assigned number of genes. 7 merged modules were identified and their corresponding assigned genes ranging from 6662 to 102. WGCNA: Weighted gene co-expression network analysis.

**Supplementary Table 4.** Gene ontology (GO) enrichment analysis of the selected hub genes in the green module under biological process (BP) category. Functional enrichment analysis was performed based on over-representation analysis (ORA) and enriched GO terms were ranked based on p-value. ID and Description are the GO identification and its name description respectively; GeneRation: no. of genes annotated to a GO term within a total number of input genes; BgRatio: no. of genes annotated to a GO term within all background genes from the database; pvalue: raw p-value; p.adjust: adjusted p-value using Benjamini-Hochberg (BH) approach; qvalue: raw q-value; Count: number of genes in the corresponding GO term.

**Supplementary Table 5.** Gene ontology (GO) enrichment analysis of the selected hub genes in the green module under molecular function (MF) category. Functional enrichment analysis was performed based on over-representation analysis (ORA) and enriched GO terms were ranked based on p-value. ID and Description are the GO identification and its name description respectively; GeneRation: no. of genes annotated to a GO term within a total number of input genes; BgRatio: no. of genes annotated to a GO term within all background genes from the database; pvalue: raw p-value; p.adjust: adjusted p-value using Benjamini-Hochberg (BH) approach; qvalue: raw q-value; Count: number of genes in the corresponding GO term.

**Supplementary Table 6.** Gene ontology (GO) enrichment analysis of the selected hub genes in the green module under cellular component (CC) category. Functional enrichment analysis was performed based on over-representation analysis (ORA) and enriched GO terms were ranked based on p-value. ID and Description are the GO identification and its name description respectively; GeneRation: no. of genes annotated to a GO term within a total number of input genes; BgRatio: no. of genes annotated to a GO term within all background genes from the database; pvalue: raw p-value; p.adjust: adjusted p- value using Benjamini-Hochberg (BH) approach; qvalue: raw q-value; Count: number of genes in the corresponding GO term.

**Supplementary Table 7.** Kyoto Encyclopedia of Genes and Genomes (KEGG) pathway analysis of the selected hub genes in the green module. Pathway analysis was performed based on over-representation analysis (ORA) and enriched pathways were ranked based on p-value. ID and Description are the pathway identification and its name description respectively; GeneRation: no. of genes annotated to a pathway within a total number of input genes; BgRatio: no. of genes annotated to a pathway within all background genes from the database; pvalue: raw p-value; p.adjust: adjusted p-value using Benjamini-Hochberg (BH) approach; qvalue: raw q-value; Count: number of genes enriched in the pathway.

**Supplementary Table 8.** Maximal Clique Centrality (MCC) score of top 15 hub genes in the green module. Connectivity of hub genes in the green module were calculated and ranked based on the corresponding MCC scores from the Cytoscape plugin CytoHubba.

**Supplementary Table 9.** Multivariate Cox regression analysis of the 5 signature genes for progression-free survival (PFS) of PCa patients. coef: regression coefficient; exp(coef): hazard ratio; se(coef): standard error; z: the Wald statistic value; Pr(>|z|): p-value of the likelihood ratio test.

**Supplementary Table 10.** Kaplan-Meier analysis of 10 selected genes from DGE and WGCNA analysis for progression-free survival (PFS) of PCa patients. pvalue: p-value of the log-rank test.

**Supplementary Table 11.** The Cambridge Prognostic Group (CPG) risk scores of PCa patients in the TCGA-PRAD training cohort. TCGA: The Cancer Genome Atlas; PRAD: prostate adenocarcinoma.

| **SampleID** | **CPG_score** |
| --- | --- |
| TCGA-HC-7075-01 | 1 |
| TCGA-HC-7080-01 | 2 |
| TCGA-HC-7748-01 | 1 |
| TCGA-HC-8259-01 | 1 |
| TCGA-HC-A8D0-01 | 2 |
| TCGA-KC-A4BN-01 | 2 |
| TCGA-2A-A8VV-01 | 1 |
| TCGA-CH-5794-01 | 2 |
| TCGA-G9-6354-01 | 2 |
| TCGA-KK-A8I5-01 | 2 |
| TCGA-SU-A7E7-01 | 4 |
| TCGA-V1-A9OH-01 | 4 |
| TCGA-VP-A879-01 | 5 |
| TCGA-CH-5746-01 | 2 |
| TCGA-CH-5750-01 | 2 |
| TCGA-CH-5767-01 | 3 |
| TCGA-CH-5771-01 | 3 |
| TCGA-CH-5790-01 | 2 |
| TCGA-EJ-5496-01 | 2 |
| TCGA-EJ-5505-01 | 3 |
| TCGA-EJ-5509-01 | 2 |
| TCGA-EJ-5514-01 | 5 |
| TCGA-EJ-5517-01 | 1 |
| TCGA-EJ-5530-01 | 2 |
| TCGA-EJ-7123-01 | 2 |
| TCGA-EJ-7218-01 | 2 |
| TCGA-EJ-7317-01 | 2 |
| TCGA-EJ-7331-01 | 2 |
| TCGA-EJ-7781-01 | 2 |
| TCGA-EJ-7782-01 | 4 |
| TCGA-EJ-7793-01 | 2 |
| TCGA-EJ-7797-01 | 2 |
| TCGA-EJ-A46D-01 | 4 |
| TCGA-EJ-A46H-01 | 2 |
| TCGA-EJ-A65G-01 | 4 |
| TCGA-EJ-A7NJ-01 | 4 |
| TCGA-EJ-A8FN-01 | 2 |
| TCGA-EJ-A8FP-01 | 4 |
| TCGA-G9-6343-01 | 3 |
| TCGA-G9-6347-01 | 1 |
| TCGA-G9-6348-01 | 2 |
| TCGA-G9-6369-01 | 3 |
| TCGA-G9-6371-01 | 1 |
| TCGA-G9-6377-01 | 2 |
| TCGA-G9-6378-01 | 2 |
| TCGA-G9-6385-01 | 2 |
| TCGA-G9-6496-01 | 3 |
| TCGA-G9-7522-01 | 2 |
| TCGA-H9-7775-01 | 2 |
| TCGA-HC-7209-01 | 1 |
| TCGA-HC-7230-01 | 2 |
| TCGA-HC-7736-01 | 2 |
| TCGA-HC-7737-01 | 2 |
| TCGA-HC-7740-01 | 2 |
| TCGA-HC-7750-01 | 2 |
| TCGA-HC-7752-01 | 2 |
| TCGA-HC-7818-01 | 2 |
| TCGA-HC-7819-01 | 4 |
| TCGA-HC-8260-01 | 2 |
| TCGA-HC-A76X-01 | 2 |
| TCGA-HC-A8D1-01 | 2 |
| TCGA-J4-8200-01 | 2 |
| TCGA-J4-A67K-01 | 3 |
| TCGA-J4-A67Q-01 | 1 |
| TCGA-J4-A67T-01 | 2 |
| TCGA-J4-A6M7-01 | 2 |
| TCGA-J4-A83J-01 | 2 |
| TCGA-J4-A83K-01 | 1 |
| TCGA-J4-A83L-01 | 2 |
| TCGA-J4-A83M-01 | 3 |
| TCGA-J4-AATV-01 | 1 |
| TCGA-J4-AAU2-01 | 1 |
| TCGA-KC-A4BL-01 | 2 |
| TCGA-KC-A7F3-01 | 2 |
| TCGA-KC-A7F5-01 | 3 |
| TCGA-KC-A7FE-01 | 2 |
| TCGA-KK-A6E2-01 | 2 |
| TCGA-KK-A6E3-01 | 2 |
| TCGA-KK-A6E8-01 | 5 |
| TCGA-KK-A7AQ-01 | 3 |
| TCGA-KK-A7AV-01 | 2 |
| TCGA-KK-A7AY-01 | 3 |
| TCGA-KK-A8IK-01 | 5 |
| TCGA-KK-A8IM-01 | 3 |
| TCGA-QU-A6IL-01 | 2 |
| TCGA-QU-A6IN-01 | 2 |
| TCGA-QU-A6IO-01 | 1 |
| TCGA-TP-A8TV-01 | 3 |
| TCGA-V1-A8MK-01 | 1 |
| TCGA-V1-A9OT-01 | 1 |
| TCGA-V1-A9OX-01 | 4 |
| TCGA-V1-A9Z9-01 | 5 |
| TCGA-V1-A9ZG-01 | 5 |
| TCGA-VN-A88L-01 | 2 |
| TCGA-VN-A88N-01 | 3 |
| TCGA-VN-A88O-01 | 2 |
| TCGA-VN-A88P-01 | 2 |
| TCGA-VN-A88R-01 | 4 |
| TCGA-VN-A943-01 | 4 |
| TCGA-VP-A878-01 | 5 |
| TCGA-VP-A87C-01 | 3 |
| TCGA-X4-A8KS-01 | 2 |
| TCGA-XA-A8JR-01 | 3 |
| TCGA-XJ-A9DK-01 | 4 |
| TCGA-XK-AAK1-01 | 3 |
| TCGA-2A-A8VO-01 | 4 |
| TCGA-2A-A8W3-01 | 5 |
| TCGA-2A-AAYU-01 | 4 |
| TCGA-CH-5739-01 | 4 |
| TCGA-CH-5752-01 | 5 |
| TCGA-CH-5765-01 | 4 |
| TCGA-CH-5766-01 | 4 |
| TCGA-CH-5768-01 | 4 |
| TCGA-CH-5772-01 | 5 |
| TCGA-CH-5791-01 | 4 |
| TCGA-CH-5792-01 | 5 |
| TCGA-EJ-5499-01 | 4 |
| TCGA-EJ-5503-01 | 5 |
| TCGA-EJ-5504-01 | 4 |
| TCGA-EJ-5516-01 | 4 |
| TCGA-EJ-5521-01 | 4 |
| TCGA-EJ-5524-01 | 5 |
| TCGA-EJ-5527-01 | 4 |
| TCGA-EJ-5531-01 | 4 |
| TCGA-EJ-5542-01 | 4 |
| TCGA-EJ-7115-01 | 4 |
| TCGA-EJ-7315-01 | 4 |
| TCGA-EJ-7318-01 | 4 |
| TCGA-EJ-7321-01 | 4 |
| TCGA-EJ-7330-01 | 4 |
| TCGA-EJ-7783-01 | 4 |
| TCGA-EJ-7785-01 | 4 |
| TCGA-EJ-8468-01 | 5 |
| TCGA-EJ-8469-01 | 5 |
| TCGA-EJ-8474-01 | 5 |
| TCGA-EJ-A46B-01 | 5 |
| TCGA-EJ-A46I-01 | 4 |
| TCGA-EJ-A65D-01 | 5 |
| TCGA-EJ-A65J-01 | 5 |
| TCGA-EJ-A7NG-01 | 4 |
| TCGA-EJ-A7NK-01 | 4 |
| TCGA-EJ-A8FO-01 | 4 |
| TCGA-EJ-A8FS-01 | 4 |
| TCGA-FC-A8O0-01 | 4 |
| TCGA-G9-6332-01 | 4 |
| TCGA-G9-6338-01 | 4 |
| TCGA-G9-6342-01 | 4 |
| TCGA-G9-6353-01 | 4 |
| TCGA-G9-6361-01 | 4 |
| TCGA-G9-6362-01 | 4 |
| TCGA-G9-6364-01 | 4 |
| TCGA-G9-6498-01 | 4 |
| TCGA-G9-7521-01 | 5 |
| TCGA-G9-7523-01 | 5 |
| TCGA-G9-A9S7-01 | 5 |
| TCGA-HC-7077-01 | 4 |
| TCGA-HC-7078-01 | 4 |
| TCGA-HC-7079-01 | 4 |
| TCGA-HC-7742-01 | 4 |
| TCGA-HC-7749-01 | 4 |
| TCGA-HC-8216-01 | 4 |
| TCGA-HC-8265-01 | 5 |
| TCGA-HI-7169-01 | 4 |
| TCGA-J4-A67L-01 | 4 |
| TCGA-J4-A67M-01 | 4 |
| TCGA-J4-A67N-01 | 4 |
| TCGA-J9-A8CK-01 | 5 |
| TCGA-J9-A8CN-01 | 4 |
| TCGA-J9-A8CP-01 | 4 |
| TCGA-KC-A4BV-01 | 5 |
| TCGA-KC-A7FA-01 | 4 |
| TCGA-KC-A7FD-01 | 4 |
| TCGA-KK-A59Y-01 | 5 |
| TCGA-KK-A6E0-01 | 5 |
| TCGA-KK-A6E6-01 | 5 |
| TCGA-KK-A6E7-01 | 5 |
| TCGA-KK-A7B2-01 | 5 |
| TCGA-KK-A8I6-01 | 4 |
| TCGA-KK-A8IF-01 | 4 |
| TCGA-KK-A8IH-01 | 4 |
| TCGA-KK-A8II-01 | 5 |
| TCGA-MG-AAMC-01 | 5 |
| TCGA-TP-A8TT-01 | 4 |
| TCGA-V1-A8MF-01 | 4 |
| TCGA-V1-A8MM-01 | 4 |
| TCGA-V1-A8MU-01 | 4 |
| TCGA-V1-A8WL-01 | 4 |
| TCGA-V1-A9O7-01 | 5 |
| TCGA-V1-A9OQ-01 | 4 |
| TCGA-V1-A9OY-01 | 4 |
| TCGA-VN-A88I-01 | 5 |
| TCGA-VN-A88K-01 | 5 |
| TCGA-VP-A872-01 | 5 |
| TCGA-VP-A875-01 | 4 |
| TCGA-VP-A87D-01 | 5 |
| TCGA-VP-A87J-01 | 4 |
| TCGA-VP-AA1N-01 | 5 |
| TCGA-XK-AAJ3-01 | 5 |
| TCGA-XK-AAJA-01 | 4 |
| TCGA-XK-AAJP-01 | 4 |
| TCGA-XK-AAJR-01 | 4 |
| TCGA-XK-AAJT-01 | 4 |
| TCGA-Y6-A8TL-01 | 4 |
| TCGA-YL-A8HO-01 | 4 |
| TCGA-YL-A8S8-01 | 5 |
| TCGA-YL-A8S9-01 | 5 |
| TCGA-YL-A8SC-01 | 5 |
| TCGA-YL-A8SO-01 | 5 |
| TCGA-YL-A9WI-01 | 5 |
| TCGA-YL-A9WL-01 | 5 |
| TCGA-ZG-A9LU-01 | 5 |
| TCGA-ZG-A9ND-01 | 5 |
| TCGA-2A-A8VX-01 | 5 |
| TCGA-4L-AA1F-01 | 5 |
| TCGA-CH-5741-01 | 5 |
| TCGA-CH-5753-01 | 5 |
| TCGA-CH-5754-01 | 5 |
| TCGA-CH-5762-01 | 4 |
| TCGA-CH-5769-01 | 5 |
| TCGA-EJ-5506-01 | 5 |
| TCGA-EJ-5507-01 | 5 |
| TCGA-EJ-5519-01 | 5 |
| TCGA-EJ-7312-01 | 4 |
| TCGA-EJ-7314-01 | 4 |
| TCGA-EJ-7325-01 | 4 |
| TCGA-EJ-7788-01 | 4 |
| TCGA-EJ-A46F-01 | 5 |
| TCGA-EJ-A65B-01 | 5 |
| TCGA-EJ-A65E-01 | 4 |
| TCGA-FC-A5OB-01 | 5 |
| TCGA-G9-6339-01 | 4 |
| TCGA-G9-6356-01 | 5 |
| TCGA-G9-6366-01 | 4 |
| TCGA-G9-6370-01 | 4 |
| TCGA-G9-6379-01 | 4 |
| TCGA-G9-A9S4-01 | 5 |
| TCGA-HC-7744-01 | 4 |
| TCGA-HC-7817-01 | 4 |
| TCGA-HC-7821-01 | 5 |
| TCGA-HC-8257-01 | 4 |
| TCGA-HC-A76W-01 | 4 |
| TCGA-HC-A9TE-01 | 5 |
| TCGA-HC-A9TH-01 | 5 |
| TCGA-HI-7168-01 | 5 |
| TCGA-HI-7171-01 | 5 |
| TCGA-J4-A6G3-01 | 5 |
| TCGA-J9-A52C-01 | 5 |
| TCGA-J9-A52E-01 | 5 |
| TCGA-J9-A8CL-01 | 5 |
| TCGA-J9-A8CM-01 | 5 |
| TCGA-KC-A4BR-01 | 5 |
| TCGA-KK-A59V-01 | 5 |
| TCGA-KK-A5A1-01 | 5 |
| TCGA-KK-A6E1-01 | 5 |
| TCGA-KK-A6E5-01 | 4 |
| TCGA-KK-A7B0-01 | 5 |
| TCGA-KK-A7B4-01 | 5 |
| TCGA-KK-A8I4-01 | 4 |
| TCGA-KK-A8I7-01 | 5 |
| TCGA-KK-A8I8-01 | 5 |
| TCGA-KK-A8IA-01 | 5 |
| TCGA-KK-A8IC-01 | 5 |
| TCGA-KK-A8ID-01 | 5 |
| TCGA-KK-A8IJ-01 | 4 |
| TCGA-KK-A8IL-01 | 5 |
| TCGA-M7-A723-01 | 4 |
| TCGA-V1-A8WV-01 | 5 |
| TCGA-V1-A8WW-01 | 5 |
| TCGA-V1-A9O9-01 | 5 |
| TCGA-V1-A9Z8-01 | 5 |
| TCGA-V1-A9ZI-01 | 5 |
| TCGA-VP-A87K-01 | 5 |
| TCGA-X4-A8KQ-01 | 5 |
| TCGA-XJ-A83G-01 | 4 |
| TCGA-XJ-A9DI-01 | 5 |
| TCGA-XJ-A9DX-01 | 5 |
| TCGA-XQ-A8TB-01 | 5 |
| TCGA-YL-A8HJ-01 | 5 |
| TCGA-YL-A8HK-01 | 5 |
| TCGA-YL-A8HL-01 | 5 |
| TCGA-YL-A8SL-01 | 5 |
| TCGA-YL-A8SP-01 | 5 |
| TCGA-YL-A8SQ-01 | 5 |
| TCGA-YL-A9WH-01 | 5 |
| TCGA-YL-A9WK-01 | 5 |
| TCGA-YL-A9WX-01 | 5 |
| TCGA-YL-A9WY-01 | 5 |
| TCGA-ZG-A8QZ-01 | 5 |
| TCGA-ZG-A9L1-01 | 5 |
| TCGA-ZG-A9L2-01 | 5 |
| TCGA-ZG-A9L4-01 | 5 |
| TCGA-ZG-A9L6-01 | 5 |
| TCGA-ZG-A9LS-01 | 5 |
| TCGA-ZG-A9LY-01 | 5 |
| TCGA-ZG-A9LZ-01 | 5 |
| TCGA-ZG-A9M4-01 | 5 |
| TCGA-ZG-A9MC-01 | 5 |
| TCGA-ZG-A9NI-01 | 5 |
| TCGA-CH-5751-01 | 5 |
| TCGA-G9-6363-01 | 5 |
| TCGA-G9-6365-01 | 5 |
| TCGA-G9-6494-01 | 5 |
| TCGA-V1-A9O5-01 | 5 |
| TCGA-V1-A9ZR-01 | 5 |

**Supplementary Table 12.** The Cambridge Prognostic Group (CPG) risk scores of PCa patients in the TCGA-PRAD testing cohort. TCGA: The Cancer Genome Atlas; PRAD: prostate adenocarcinoma.

| **SampleID** | **CPG_score** |
| --- | --- |
| TCGA-HC-7210-01 | 2 |
| TCGA-HC-7212-01 | 2 |
| TCGA-QU-A6IM-01 | 2 |
| TCGA-QU-A6IP-01 | 1 |
| TCGA-2A-A8VL-01 | 1 |
| TCGA-KK-A6E4-01 | 2 |
| TCGA-VP-A87B-01 | 4 |
| TCGA-2A-AAYO-01 | 1 |
| TCGA-CH-5737-01 | 3 |
| TCGA-CH-5740-01 | 2 |
| TCGA-CH-5743-01 | 2 |
| TCGA-CH-5744-01 | 3 |
| TCGA-EJ-5497-01 | 2 |
| TCGA-EJ-5498-01 | 3 |
| TCGA-EJ-5502-01 | 3 |
| TCGA-EJ-5510-01 | 3 |
| TCGA-EJ-5512-01 | 2 |
| TCGA-EJ-5522-01 | 2 |
| TCGA-EJ-5525-01 | 5 |
| TCGA-EJ-7125-01 | 2 |
| TCGA-EJ-7784-01 | 3 |
| TCGA-EJ-7786-01 | 2 |
| TCGA-EJ-7791-01 | 2 |
| TCGA-EJ-7792-01 | 2 |
| TCGA-EJ-7794-01 | 2 |
| TCGA-EJ-A65M-01 | 1 |
| TCGA-EJ-A7NF-01 | 2 |
| TCGA-EJ-A7NH-01 | 2 |
| TCGA-EJ-AB20-01 | 1 |
| TCGA-EJ-AB27-01 | 1 |
| TCGA-G9-6329-01 | 2 |
| TCGA-G9-6333-01 | 3 |
| TCGA-G9-6351-01 | 2 |
| TCGA-G9-7509-01 | 1 |
| TCGA-G9-7519-01 | 2 |
| TCGA-G9-7525-01 | 3 |
| TCGA-H9-A6BX-01 | 1 |
| TCGA-HC-7211-01 | 3 |
| TCGA-HC-7747-01 | 2 |
| TCGA-HC-8258-01 | 1 |
| TCGA-HC-8262-01 | 4 |
| TCGA-HI-7170-01 | 1 |
| TCGA-J4-A67O-01 | 2 |
| TCGA-J4-A67R-01 | 3 |
| TCGA-J4-A83N-01 | 2 |
| TCGA-KC-A7F6-01 | 2 |
| TCGA-KK-A59Z-01 | 3 |
| TCGA-KK-A6DY-01 | 2 |
| TCGA-V1-A8ML-01 | 2 |
| TCGA-V1-A8WN-01 | 1 |
| TCGA-V1-A8X3-01 | 2 |
| TCGA-V1-A9OF-01 | 1 |
| TCGA-VN-A88Q-01 | 4 |
| TCGA-VP-A876-01 | 4 |
| TCGA-VP-A87E-01 | 1 |
| TCGA-WW-A8ZI-01 | 4 |
| TCGA-XJ-A83H-01 | 2 |
| TCGA-XK-AAIR-01 | 4 |
| TCGA-XK-AAJU-01 | 3 |
| TCGA-2A-A8W1-01 | 4 |
| TCGA-2A-AAYF-01 | 4 |
| TCGA-CH-5745-01 | 4 |
| TCGA-CH-5763-01 | 4 |
| TCGA-CH-5789-01 | 4 |
| TCGA-EJ-5508-01 | 4 |
| TCGA-EJ-5511-01 | 4 |
| TCGA-EJ-5515-01 | 4 |
| TCGA-EJ-5526-01 | 5 |
| TCGA-EJ-5532-01 | 4 |
| TCGA-EJ-8472-01 | 5 |
| TCGA-EJ-A46G-01 | 5 |
| TCGA-G9-6367-01 | 5 |
| TCGA-G9-6384-01 | 4 |
| TCGA-G9-6499-01 | 5 |
| TCGA-G9-7510-01 | 5 |
| TCGA-HC-A8CY-01 | 5 |
| TCGA-J4-A67S-01 | 4 |
| TCGA-J4-A6G1-01 | 5 |
| TCGA-J4-A83I-01 | 4 |
| TCGA-J4-AATZ-01 | 5 |
| TCGA-J9-A52D-01 | 5 |
| TCGA-KK-A7AW-01 | 4 |
| TCGA-KK-A7B1-01 | 4 |
| TCGA-KK-A8I9-01 | 5 |
| TCGA-KK-A8IG-01 | 4 |
| TCGA-XJ-A83F-01 | 4 |
| TCGA-YL-A8SB-01 | 5 |
| TCGA-YL-A8SH-01 | 4 |
| TCGA-YL-A8SI-01 | 5 |
| TCGA-YL-A8SR-01 | 5 |
| TCGA-ZG-A9LM-01 | 5 |
| TCGA-CH-5764-01 | 4 |
| TCGA-CH-5788-01 | 4 |
| TCGA-EJ-5495-01 | 5 |
| TCGA-EJ-5501-01 | 4 |
| TCGA-EJ-7327-01 | 4 |
| TCGA-EJ-A65F-01 | 5 |
| TCGA-EJ-A6RA-01 | 5 |
| TCGA-EJ-A7NM-01 | 5 |
| TCGA-EJ-A7NN-01 | 4 |
| TCGA-FC-A66V-01 | 4 |
| TCGA-G9-A9S0-01 | 5 |
| TCGA-HC-7213-01 | 5 |
| TCGA-J4-8198-01 | 4 |
| TCGA-J9-A52B-01 | 5 |
| TCGA-KK-A7AU-01 | 5 |
| TCGA-KK-A7B3-01 | 5 |
| TCGA-V1-A8MG-01 | 4 |
| TCGA-V1-A8WS-01 | 4 |
| TCGA-V1-A9OA-01 | 5 |
| TCGA-V1-A9OL-01 | 5 |
| TCGA-V1-A9Z7-01 | 5 |
| TCGA-V1-A9ZK-01 | 5 |
| TCGA-VP-A87H-01 | 5 |
| TCGA-XK-AAIV-01 | 5 |
| TCGA-XK-AAIW-01 | 5 |
| TCGA-YJ-A8SW-01 | 5 |
| TCGA-YL-A8HM-01 | 5 |
| TCGA-YL-A8SA-01 | 5 |
| TCGA-YL-A8SJ-01 | 5 |
| TCGA-YL-A8SK-01 | 5 |
| TCGA-YL-A9WJ-01 | 5 |
| TCGA-ZG-A9LB-01 | 5 |
| TCGA-ZG-A9LN-01 | 5 |
| TCGA-ZG-A9N3-01 | 5 |
| TCGA-2A-A8VT-01 | 5 |
| TCGA-EJ-5518-01 | 5 |
